# The gastrointestinal electrical mapping suite (GEMS): software for analyzing and visualizing high-resolution (multi-electrode) recordings in spatiotemporal detail

**DOI:** 10.1186/1471-230X-12-60

**Published:** 2012-06-06

**Authors:** Rita Yassi, Gregory O’Grady, Nira Paskaranandavadivel, Peng Du, Timothy R Angeli, Andrew J Pullan, Leo K Cheng, Jonathan C Erickson

**Affiliations:** 1 Auckland Bioengineering Institute, The University of Auckland, Auckland, New Zealand; 2 Department of Surgery, The University of Auckland, Auckland, New Zealand; 3 Riddet Institute, Auckland, New Zealand; 4 Department of Engineering Science, The University of Auckland, Auckland, New Zealand; 5 Department of Surgery, Vanderbilt University, Vanderbilt, TN, USA; 6 Department of Physics-Engineering, Washington & Lee University, Lexington, VA, USA; 7 Department of Surgery, The University of Auckland, Private Bag 92019, Auckland, New Zealand

**Keywords:** Slow wave, Spike, Signal processing, Electrophysiology, Motility, Tachygastria

## Abstract

**Background:**

Gastrointestinal contractions are controlled by an underlying bioelectrical activity. High-resolution spatiotemporal electrical mapping has become an important advance for investigating gastrointestinal electrical behaviors in health and motility disorders. However, research progress has been constrained by the low efficiency of the data analysis tasks. This work introduces a new efficient software package: GEMS (Gastrointestinal Electrical Mapping Suite), for analyzing and visualizing high-resolution multi-electrode gastrointestinal mapping data in spatiotemporal detail.

**Results:**

GEMS incorporates a number of new and previously validated automated analytical and visualization methods into a coherent framework coupled to an intuitive and user-friendly graphical user interface. GEMS is implemented using MATLAB®, which combines sophisticated mathematical operations and GUI compatibility. Recorded slow wave data can be filtered via a range of inbuilt techniques, efficiently analyzed via automated event-detection and cycle clustering algorithms, and high quality isochronal activation maps, velocity field maps, amplitude maps, frequency (time interval) maps and data animations can be rapidly generated. Normal and dysrhythmic activities can be analyzed, including initiation and conduction abnormalities. The software is distributed free to academics via a community user website and forum (http://sites.google.com/site/gimappingsuite).

**Conclusions:**

This software allows for the rapid analysis and generation of critical results from gastrointestinal high-resolution electrical mapping data, including quantitative analysis and graphical outputs for qualitative analysis. The software is designed to be used by non-experts in data and signal processing, and is intended to be used by clinical researchers as well as physiologists and bioengineers. The use and distribution of this software package will greatly accelerate efforts to improve the understanding of the causes and clinical consequences of gastrointestinal electrical disorders, through high-resolution electrical mapping.

## Background

Gastric peristalsis is coordinated by an underlying electrical activity, termed slow waves, which are generated and propagated by the interstitial cells of Cajal (ICC) [1]. Disordered slow wave activity has long been associated with several gastric motility disorders, including gastroparesis and functional dyspepsia [2,3], however, the functional significance of gastric electrical abnormalities remains a focus of debate and research. Clinical interest in the evaluation of slow wave activity has recently been renewed by strong evidence linking ICC network pathology with motility disorders including gastroparesis [4].

Multi-electrode, high-resolution (HR) mapping has become a key advance for studying gastrointestinal (GI) slow wave behaviors. This method involves the placement of spatially-dense arrays of many electrodes over a gut segment, in order to record electrical activation sequences simultaneously at multiple points. Activation time maps (graphic and quantitative characterizations of electrical propagation) can be generated from these recordings, eliciting detailed information on the spatiotemporal patterns of electrical propagation [5], which cannot be derived from alternative techniques, such as sparse electrode recordings or cutaneous electrogastrography (EGG).

The value and potential of the spatiotemporal data provided by HR mapping has been demonstrated in several recent studies that have applied the technique to establish new descriptions of normal slow wave activation in large animal models and humans [6-8], and to define new tissue-level mechanisms of gastric dysrhythmia [9,10]. Clinical and therapeutic translation is now progressing [11,12].

A critical barrier to research progress in gastrointestinal HR mapping has been the laborious nature of the data processing, which has typically been performed manually. A vast volume of data is typically recorded in multi-electrode mapping studies, with several thousand slow wave signals potentially being recorded in each experiment for assessment. Recently, however, significant methodological advances have been presented that serve to improve the analysis efficiency of GI slow wave mapping and expand its applications. These methods include new algorithms to automatically identify slow wave activation times [13], partition slow waves into individual cycles, to draw spatiotemporal maps [14], and to calculate velocities [15].

In the cardiac field, where multi-electrode mapping has been practiced for several decades, comparable methods have long been integrated into software frameworks that have been widely used (e.g. see [16-18]). These frameworks are critical, because they allow the key experimental and clinical results to be rapidly and accurately extracted from the vast sets of raw data. Commercial systems are also now available for cardiac mapping, such as the CARTO and NavX systems, which are routinely used in clinical practice [19].

Cardiac mapping software cannot simply be applied to GI data due to the different signal characteristics of the electrical events and their propagation patterns during both normal and abnormal activation [13,14]. In the GI field, until now, the only existing software system for analyzing electrical mapping data has been ‘SmoothMap’ [20], which has been fundamental to enabling most HR GI mapping studies to date (e.g., [6-9]). However, there are a number of important limitations with SmoothMap, which provided motivation to develop an alternative or complimentary software framework for GI mapping analysis. Most significantly, several laborious analysis tasks must still be performed manually in SmoothMap, such as activation time identification, cycle partitioning, and isochronal mapping. In addition, the ongoing need to establish new analytical methods in this emerging field prompted us to establish an extensible analysis system within a standard technical computing language that could continue to be augmented in future by a user community.

To this end, a new GI electrical mapping software is presented. The software is designed to be used by non-experts in data and signal processing, and is intended to be used by clinical researchers as well as physiologists and bioengineers. Technical knowledge is, however, likely to be of additional value for tuning specific parameters that optimize the performance of the software. Importantly, this software is a research tool, and is not intended for clinical application in its present form.

## Implementation

A user friendly graphical user interface (GUI) package, termed the Gastrointestinal Electrical Mapping Suite (GEMS) was implemented to facilitate and accelerate GI multi-electrode data analysis. The package incorporates algorithms for importing and filtering data, automatically detecting slow wave activation times, clustering individual waves into wavefronts, and calculating wavefront time intervals (frequencies), velocity and amplitude profiles. In addition, the package also allows the user to rapidly generate high-quality HR maps of the activation times, velocity fields, amplitudes and time-intervals with minimal manual labor.

### Package overview and architecture

GEMS is implemented using MATLAB® R2009a (The MathWorks Inc., Natick, MA, USA), a licensed program which combines sophisticated mathematical operations and GUI compatibility. The benefits associated with using MATLAB include ease of use, in-built libraries of mathematical and graphical routines, and the ability to run on several operating systems (MS-Windows, Linux, and Mac OS X). GEMS can be run as a stand alone application that does not require the user to own a Matlab license.

**Figure 1:**
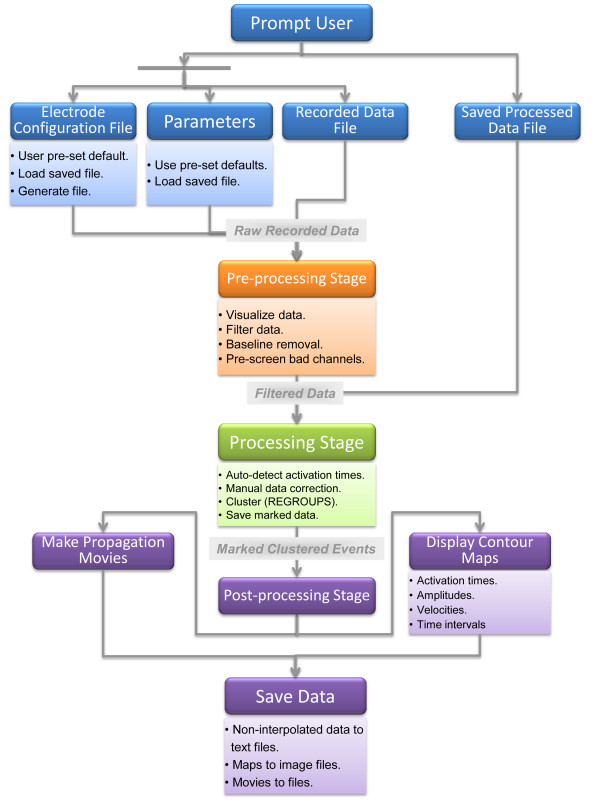
**GEMS architecture.** GEMS is structured in three stages; pre-processing, processing and post-processing. In the pre-processing stage, the user can visualize and filter the recorded raw data. Electrodes with no reliable recorded data can be discarded at this stage. In the processing stage, activation times can be automatically detected using the in-built algorithms and the marked events can be partitioned and grouped/clustered into a series of wavefronts/cycles. The final stage is the post-processing stage. In this stage, high-resolution maps can be produced to show the propagation and distribution of activation times, velocity fields, and amplitudes, and propagation movies can also be generated.

Data analysis in GEMS is divided into three stages: pre-processing, processing and post-processing as shown in Figure1. In the pre-processing stage, the ‘raw recorded data’ input is converted to a file that is visualized and filtered in MATLAB. Channel selection controls allow the user to discard electrodes that contain no reliable recorded data (e.g., due to poor contact of the electrode with the GI tract or a technical fault). The output of the pre-processing stage is the ‘filtered data’, which becomes the input for the processing stage. Using the inbuilt algorithms, activation times can be automatically detected and the marked events can be grouped or ‘clustered’ into a series of wavefronts. The output of the processing stage is ‘marked clustered events’, which becomes the input for the final post-processing stage. In this stage, pseudo-colored contour maps can be produced to show the propagation and distribution of activation times, amplitudes and velocity fields. In addition, propagation movies can be produced to allow animated visualization of the spread of electrical activity.

At each stage, the user can interact with the program, perform processing steps, and tune multiple parameters to their needs via a user-friendly GUI. Further explanations of each stage of the software implementation are now provided in detail.

### Input requirements

Upon launching GEMS, the user is prompted to provide a file containing the recorded raw electrical data, or a previously saved file. GEMS is currently configured to work with the ActiveTwo System (BioSemi, Amsterdam), which generates data files in a 24 bit version of the European Data Format (.bdf). However, GEMS could also be readily configured to work with a number of other acquisition systems producing a file that can be imported into MATLAB, as per the needs of the user community. Signal files in simple text format can be imported.

### Electrode configuration file

The distribution and inter-electrode spacing of the electrodes in the recording array must be specified in order to generate maps of the propagation of the electrical activity along and around the GI tract. This information is stored in an appropriately formatted file (‘the electrode configuration file’) for input, or standard templates can be generated from within GEMS. During the pre- and processing stages, the user can view and orient the electrode configuration to match the experimental positioning of their array on the GI tract to aid visualization of the processed data.

### Parameters file

GEMS uses a large set of parameters for both the GUI and the back-end algorithms. These parameters cover a substantial range of functions, including filtering methods, the “Falling-Edge, Variable Threshold” (FEVT) and “Region Growing using Polynomial Surface Stabilization” (REGROUPS), algorithm tuning options (discussed below), activation time map design (e.g., isochronal intervals, plotting of an electrode grid, contour smoothing and use of interpolation), and propagation animation settings (e.g., start and end times, wavefront colors, supplementary text).

Technical knowledge of the algorithms used by GEMS (Table1) is helpful in order to tune the parameters for optimal software performance and graphical outputs. For example, modifying parameters for event detection and clustering (see below), according to the profiles of individual data sets, can enable more accurate results in response to particular types of noise or dysrhythmic events [13,14]. However, a default set of parameters that is suitable for typical gastric applications has been predefined in the software, meaning that the software should also be readily useable by researchers not familiar with data or signal processing principles.

**Table 1:** Key Algorithms Employed in GEMS and Quantitative Validation References

**Algorithm**	**Function**	**Validated In:**
*Filtering: moving median, wavelet, Butterworth and Savitsky-Golay filters*	Reduce baseline drift and high-frequency noise from signals.	Paskaranandavadivel 2011 [21]
*Falling-Edge Variable Threshold (FEVT)*	Automated marking of slow wave activation times	Erickson et al. 2010 [13]
*Region-GRowing Using Polynomial-surface- estimate Stabilization (REGROUPS)*	Automated clustering (grouping) of slow wave activation times into discrete cycles	Erickson et al. 2011 [14]
*Spatial Visualization and Interpolation (SIV) Scheme*	Automated interpolation and graphing of isochronal maps of slow wave propagation patterns	Erickson et al. 2011 [14]
*Amplitude Estimation by Peak-Trough Zero-Crossings Scheme*	Automated estimation of extracellular amplitudes	Paskaranandavadivel 2011 [21]
*Velocity Estimation by Smoothed Finite Difference Scheme*	Automated estimation of velocity fields for slow wave propagation maps	Paskaranandavadivel 2012 [15]
*Amplitude and Velocity Field Visualization Schemes*	Automated graphing of spatial data on amplitude and propagation velocity	Paskaranandavadivel 2011,2012 [15,21]

A ‘parameters GUI’ is incorporated into GEMS, which groups all of the parameters systematically according to their functions. This GUI allows the user to alter the parameters, save them, and also load alternative previously defined parameter sets. A brief description of each parameter is displayed next to each option.

### Algorithms

The core back-end analysis functions within GEMS are comprised of slow wave analysis algorithms that span the analysis process from slow wave event detection to graphical visualization. Brief descriptions of these algorithms are provided in the following sections, and they are listed in Table1 together with their validation references. GEMS is readily extensible such that new analysis algorithms developed by the user community can be readily incorporated.

### Filters

Filters can be applied to the gastric bioelectrical signals to eliminate baseline wander (such as body movement artifacts) and high frequency noise (such as power line interference) [21,22]. A range of filter types are available, ranging from low pass, high pass, band pass, and averaging filters. The filters that are currently implemented in GEMS are Butterworth, Wavelet, Savitzky Golay and Moving Median filters [23-25]. Recent work suggests that the moving median and Savitzky-Golay filters are among the most appropriate filters for many mapping purposes [21]. Users can adjust the filter parameters as required.

### Activation time detection

As in cardiac mapping, a fundamental step in GI mapping is the detection of the biphasic or triphasic slow wave depolarization events, which approximate the second derivative of the transmembrane potential, and correspond to the arrival of the depolarization wavefront at the region sensed by the electrode [13]. Determining these activation times is the first essential step in the mapping process, and must be achieved to generate isochronal, velocity, amplitude and time-interval maps from the HR data.

The FEVT (Falling-Edge, Variable Threshold) algorithm automates detection of slow wave activation times in GEMS. The FEVT algorithm identifies relatively high-energy, high-frequency, downward deflecting components in the pre-processed recordings. FEVT is described in detail with its validation in [13]. When the signal under analysis crosses the time-varying threshold, a slow wave event is marked with a red point. The FEVT algorithm increases data processing speed by ~100x compared to manual marking, while maintaining high sensitivity (~90%) and low false-negative and false-positive rates (~10%), even when the recorded signal-to-noise ratio is relatively low [13,26].

### Clustering algorithm

The second essential step in the mapping process is to ‘cluster’ the many individually marked activation times into groups that represent independent slow wave cycles (i.e., such that all data points in a clustered group relate to the same propagating wavefront). The REGROUPS (Region Growing using Polynomial Surface Stabilization) method is used to cluster activation times in GEMS (described in detail with its validation in [14]).

The REGROUPS algorithm uses a recursive search technique in combination with a continuously updated 2^nd^ order spatiotemporal filter. The marked activation times are searched and the algorithm predicts when activation times should occur at adjacent electrodes. Once the activation times are chosen, they are grouped into ‘cycles’ or wavefronts. REGROUPS has been shown to properly group activation times from normal and abnormal propagation patterns, including when data are of relatively patchy quality [14]. However, manual correction may be required in complex cases (see below).

### Representing activation times

Producing contour maps of activation times (isochronal) is a highly effective means of displaying a large volume of data in an intuitive graphical display that can be readily interpolated and understood [27]. In an isochronal map, contour lines are drawn at or between electrode points where depolarization occurred at the same time, and the successive contour lines (and intervening pseudo-colored ‘isochronal bands’) quantify the propagation sequence.

A two-step ‘Spatial Interpolation and Visualization’ (SIV) scheme is implemented in GEMS to aid visualization of slow wave patterns to account for patchy data quality when necessary [14]. The user is presented with the option to interpolate between data values. In brief, if a blank site (no marked activation times) is surrounded by a threshold number of marked sites, then the blank site’s activation time is interpolated. The second stage repeats a similar process, this time using marked sites interpolated during the first stage, as well as the initially marked sites to fill in the missing borders. This scheme was implemented due to its practicality and simplicity. Full details of the SIV scheme and its validation are given in [14].

### Velocity and amplitude calculation

The velocity and amplitude of the propagating wavefronts can be estimated/computed using GEMS. These computations provide valuable spatial and statistical data on regional variations in the speed, direction and extracellular amplitude of slow wave propagation from cycle to cycle, which are important variables for understanding slow wave excitation in health and disease. In the normal human stomach, for example, HR mapping has recently shown that normal slow wave excitation is characterized by marked velocity and amplitude transitions occuring between the normal pacemaker region and the corpus, and within the gastric antrum [8]. Abnormally high velocity and amplitude activity has also recently been found to routinely accompany gastric dysrhythmias, and the velocity and amplitude data provided by GEMS can, therefore, assist in identifying and understanding the onset, location and pattern of abnormal events [28,29].

The default method for velocity field calculation in GEMS is a finite difference approach that incorporates interpolation and Gaussian filter smoothing functions [15]. According to a recent validation study, this velocity calculation method is superior to alternative approaches that have been used previously in the GI field, such as simple finite differences [1,5], and polynomial-based methods [11,30], because it has a low average error and aids accurate visualization of velocity fields [15]. The above two alternative approaches are also available for use in GEMS, however, if desired by the user.

The amplitude of the gastric signals is obtained by taking a 1.5 s window of the signal for analysis, based on the point of interest (detected by FEVT). In the section of the signal considered for analysis, the amplitude of the gastric signal is estimated using a peak-trough detection algorithm using the ‘zero-crossing’ of the first and second order signal derivative [21,31]. This approach is superior to simple peak-trough detection algorithms, as commonly used previously in the GI HR mapping field [5,20], because it better accounts for competing noise and slow wave fractionation, thereby improving accuracy [21].

The magnitude of the amplitudes and velocities are represented with colors at associated electrode points, and the directions of the velocity field are displayed as arrows overlaid at the electrode points on the maps [15,21]. Examples of amplitude and velocity map generation and output are provided in the following section.

### Time-interval (frequency) calculation

GEMS also incorporates algorithms for calculating the frequency of wavefronts. To achieve this step, GEMS calculates the intervals between activation times recorded by each electrode in the array between consecutive cycles [7,8]. These outputs can be viewed as ‘time-interval maps’, which show the spatial pattern of the time intervals between consecutive cycles, together with the mean and standard deviation values. These features enable ‘bradygastric’ (reduced slow wave frequency) and ‘tachygastric’ (rapid slow wave frequency) dysrhythmic events to be detected, for correlation with the propagation patterns revealed by the isochronal and velocity field maps.

## Results

### Pre-processing stage

**Figure 2:**
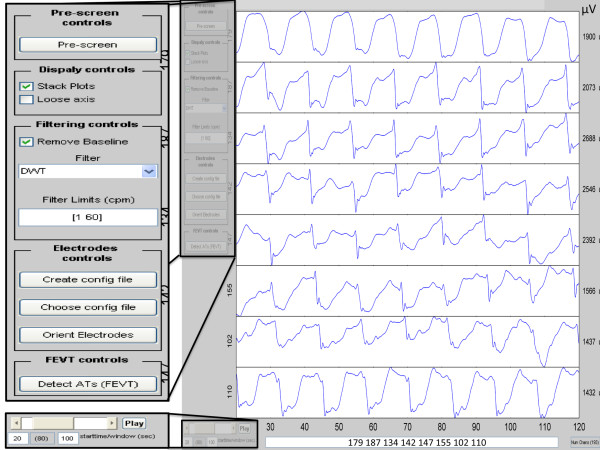
**Pre-processing stage.** The raw recorded electrical signals are shown for the corresponding electrodes listed in the box at the bottom of the figure (electrodes 179, 187, 134, 142, 147, 155, 102, and 110). The number of the electrode associated with each trace is displayed to the left of each signal plot. On the right hand side the maximum amplitude (i.e., maximum value - minimum value) is displayed in μV. On the left hand side of the figure, users can use the available options to filter and remove baseline drift from the raw data. On the bottom left hand side of the figure, users can select the time period (start time and duration) for data analysis. In this example the starting time is 20s and 100s display time duration.

Once the raw data file is read into GEMS, the user is presented with the pre-processing stage display as shown in Figure2. In this stage, the recorded data can be viewed and filtered using a GUI. The user can view the recorded data from each electrode individually, or from multiple electrodes at a time as a stacked plot. The duration of the viewed data can be altered to show a narrow time window of events in finer detail, or a wider time window in coarser detail.

The main purpose of the pre-processing stage is to perform signal processing tasks, such as removal of baseline drift and noise. A range of filter options are currently available to the user to fulfill these tasks (detailed above), the effects of which are displayed live in the pre-processing stage. The user can also mark electrodes that demonstrate a poor signal-to-noise ratio, or no signal, such that they will not be considered for activation time detection. These channels can otherwise induce false positives into the FEVT algorithm results, leading to inaccuracies in the mapping outcomes [13]. New methods are currently being developed to automatically detect channels with poor signal quality, allowing them to be deleted prior to further data processing [32].

### Processing stage

The data prepared in the pre-processing stage is passed into the processing stage, incorporating the channel numbers, time window, baseline removal, filtering methods and other parameter settings specified by the user. Slow wave activation time marking occurs automatically via the FEVT method [13]. The default FEVT parameters that are implemented have been optimized for the processing of gastric signals; a user wishing to tune these parameters further can access them conveniently through the parameter selection window. Parameters for FEVT processing of small intestine slow wave signals are also currently being identified [33].

### Electrode selection and activation time reviewing

The automatically marked data are viewed within a new window, where they are manually reviewed for further processing. The false positive and negative rates of FEVT depend on the quality of the input raw data, filtering methods and the choice of tuning parameters [13]. Considering the large quantity of recorded data, it is essential that the user can review the marks and manually correct these irregularities in the easiest and most efficient way possible.

**Figure 3:**
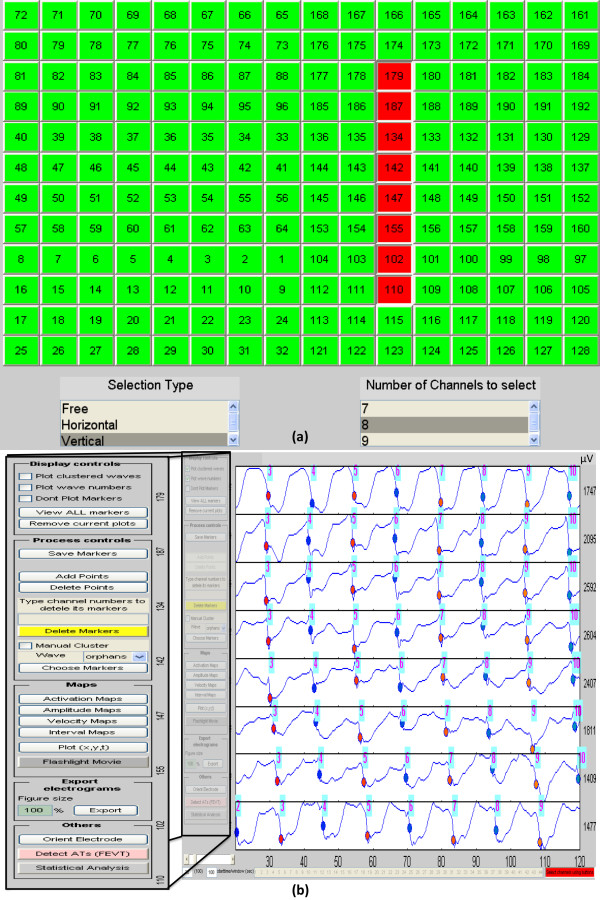
**Processing marked data.** (a) shows the electrode configuration of a sample file in a form of buttons. The user can display the marked events of one or multiple electrodes using the ‘*Selection Type’* and ‘*Number of Channels to Select’* list boxes. In this example the user has chosen to select a vertical column of 8 channels and clicked on channel 179. Based on the selection made in (a) the processed data of the corresponding electrodes are then shown in (b). Using the controls on the left hand side of figure (b), the user can add/delete markers on the signals of the selected electrodes, view the clustered events and manually group/re-group the marked events. In this example the marked events shown with different colored circles on the signals have already been grouped into different wave numbers as shown with the numbers on top of each of the marked events. Waves 3–10 at electrodes 179, 187, 134, 142, 147, 155, 102, and 110 are shown in (b).

One important feature of the GUI is, therefore, providing the user with the flexibility to visually inspect the signals, select single or multiple electrode/s, and modify the marked events with a mouse click. This method emulates the approach that is used in SmoothMap and several cardiac mapping systems [18]. In this approach, adjacent electrode channels can be viewed as a stacked column of electrograms, to visually evaluate mark quality and the ‘time lag’ between activation times in adjacent channels that is a hallmark of propagating activation sequences. Figure3(a) shows the GEMS ‘channel selection’ window, comprising an example electrode configuration matrix displayed in the form of buttons. The user can select electrodes to view, by row, column, or free choice, and the selected electrode signals are then displayed in a stacked column in a separate figure for editing, as per the example in Figure3(b)*.* Using the processing buttons on the left hand side of the GUI, shown in Figure3(b) the user can delete or add activation time marks from the displayed electrodes using a cursor target.

Each electrogram in the processing window is scaled to its amplitude range, which is displayed next to the signal. The electrograms can also be displayed according to a common amplitude scale, and exported as a high-quality image of a size specified by the user.

### Cycle partitioning

Once the activation time marks are prepared, they can be automatically clustered into common wavefronts (‘cycles’) using the REGROUPS algorithm outlined above [14]. An alternative or complimentary manual clustering option is also available, which is useful for correcting abnormalities that can occur when REGROUPS is applied to data of poorer quality, of highly atypical or abnormal pattern that REGROUPS cannot adequately handle, or when the user wishes to view an alternate clustering option.

Using the processing and display controls the user can review the REGROUPS results in stacked electrograms via the same channel selection method described above as shown in Figure3(b). Clustered cycle numbers and markers are uniquely colored by cycle number to guide visualization of the clustered results. Ungrouped events, termed ‘orphans’, are marked as green squares without numbers. These orphans may represent isolated activities, FEVT false positives, or REGROUPS false negatives, and can be manually distributed into numbered wave clusters if desired by the user.

### Post-processing stage

Once the data are processed to the user’s satisfaction, the user is able to generate data maps, tables, figures and movies to provide visual interpretation of the experimental data and use them for publication or presentation purposes. A detailed description of the different visual options and their underlying algorithms is given in the following sections.

### Activation time contour mapping

As detailed above, activation time (isochronal) maps are often among the most valuable results derived from multi-electrode mapping studies, conveying information about the pattern, direction, speed and variability of propagation [27]. Activation maps for clustered marked events are generated in GEMS according to the parameters specified by the user (e.g., isochronal spacing, interpolation methods and color range).

**Figure 4:**
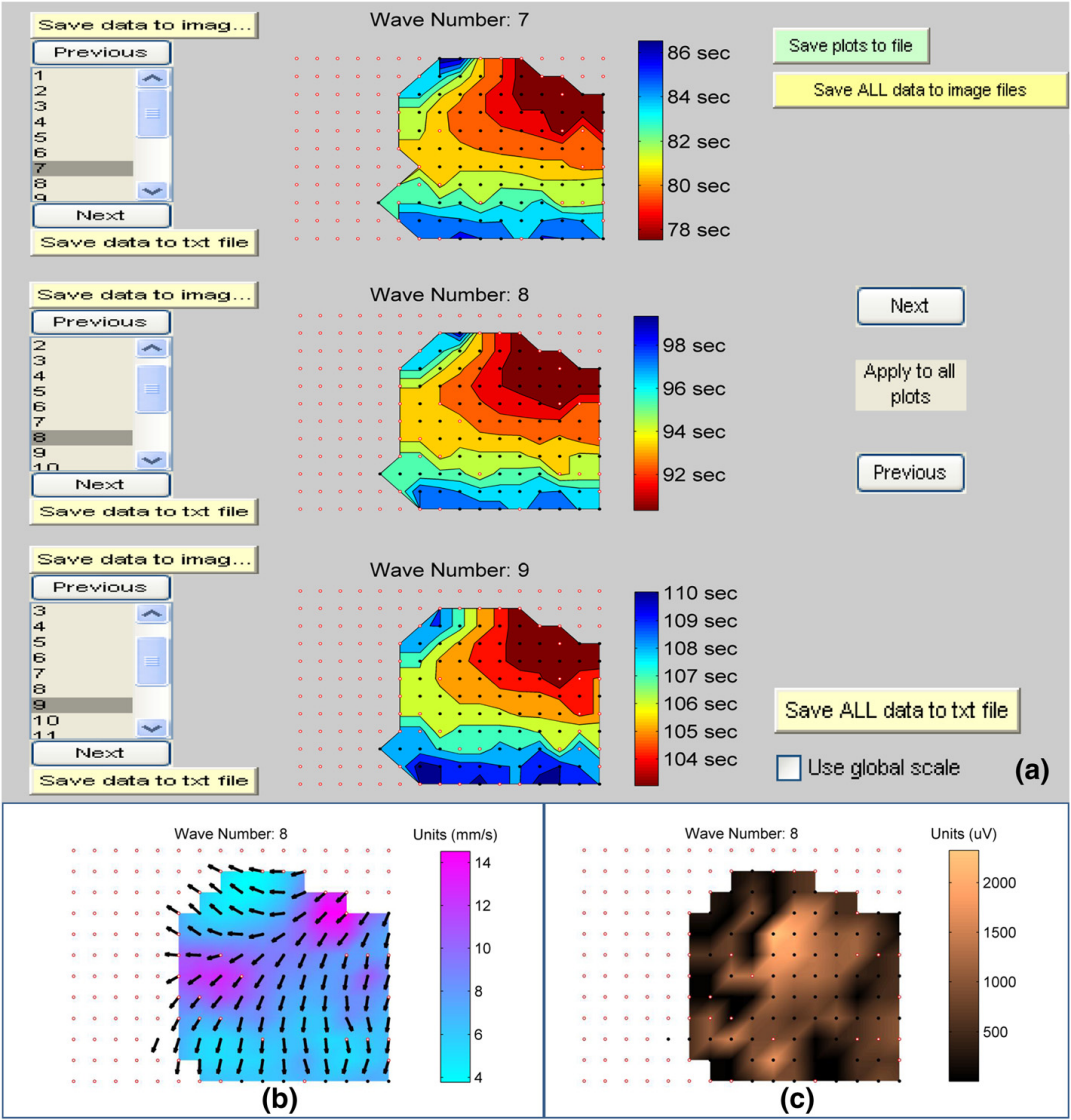
**High-resolution maps.** The propagation of the marked waves along the matrix of the electrodes can be displayed using high-resolution maps; (a) shows the isochronal activation time maps for waves 7, 8 and 9. (b) and (c) illustrate examples of velocity and amplitude maps for wave 8. The user can select which wave number to view on each plot in (a) using the list boxes associated with each plot on the left hand side of figure (a). The color scheme for the velocity maps in (b) show the magnitude of the velocity while the arrows show the direction of the velocity along the electrode matrix. The displayed maps can be exported to high-resolution image files (b and c are samples of the exported files) for publication or presentation purposes using the ‘*Save data to image file’* buttons shown on the left hand side of figure (a). The numeric values associated with the maps can be exported to text files for further statistical analysis using the ‘*Save data to text file’* buttons shown on the left hand side of figure (a).

GEMS plots the identified activation times in the spatial arrangement determined by the electrode configuration file. Either simple ‘patch plots’ of the activation times or contoured smoothed isochronal maps can be plotted. If contoured plots are used, the time bands between the isochronal bars are pseudo-colored according to a red-blue spectrum, as per the example shown in Figure4(a)*.* The electrode array can also be superimposed on the map as a grid of circles; black circles indicate electrodes with detected marked events, and white circles indicate electrodes with no detected events (including where data were interpolated according to the algorithm described above) [14].

### Amplitude, velocity and time-interval mapping

In addition to viewing the activation time maps, users can also view spatial maps of the amplitudes, velocities and time intervals (frequencies) of the detected slow waves comprising each wavefront. These data are generated according to the algorithms described above. For the velocity fields, arrows represent direction of propagation and are overlaid on a pseudo-colored ‘speed map’ Figure4(b). For the amplitude map, a pseudo-color is used to represent the magnitude of the wave at each electrode site Figure4(c). Time-interval maps are displayed as ‘patch plots’.

### Data export

For all of the different map types, GEMS is designed to allow the user to view three wavefronts at a time as illustrated in Figure4(a)*,* and the user can navigate between the different waves. This feature allows the user to compare a current wavefront with the previous and successive wavefronts. The plotted maps can be saved into high-quality image files for the purpose of analysis, publications and presentation. Examples of results from GEMS can be found in a recent publication, including maps of dysrhythmic slow wave patterns [10].

In addition, the values of the activation times, time intervals, amplitudes and velocities of the selected events can be saved to text files for statistical analysis (i.e., non-interpolated data only). Marked data can also be exported in a text file format suitable for import into SmoothMap.

### Propagation movies

Movies are used to visualize the propagation of the marked events along the electrode matrix as a function of time, and are a particularly useful aid for understanding or presenting complex data sequences [9,27]. Activation times are colored in sequence over an array that is arranged in the same manner as the electrode configuration file. The colored pixels are then set to fade before turning off again to simulate visualizations of the wavefront’s ‘refractory tail’. Wavefront sequences can be animated according to the clustered or unclustered times. If clustered times are used, then each successive wavefront can be uniquely colored to improve clarity of visualization. The user chooses the start and end times of the desired recording period, and can set the frame rate and duration of the displayed ‘tailing edge’. The propagation movies can be exported as per the example in Additional file 1. In addition to the saved files, a movie player is generated to allow the user to scroll through the files, pause, fast forward, loop, etc. Examples of movies of dysrhythmic activities generated through GEMS can be found in a recent publication that employed the software [10].

### Data saving and re-loading

GEMS allows the user to save the filtered data with the marked events (clustered or unclustered). The file will also automatically save the electrode configuration and the default parameters used to obtain the results. Alternatively, the user can also save the electrode configuration and the default parameters into isolated files for the flexibility of using them with other experiments. To reload analyzed data, at start-up the user can specify the analyzed data file and GEMS will launch in the processing mode.

### System application

The GEMS Software has undergone both quantitative validation (detailed in Table1), and qualitative validation. Qualitative validation has been recently demonstrated in a published experimental study in a porcine model, in which a range of gastric dysrhythmias were characterized, quantified, and classified, including several new patterns, such as rapid slow wave re-entry in the gastric corpus [10]. Further clinical works are currently progressing [12].

**Figure 5:**
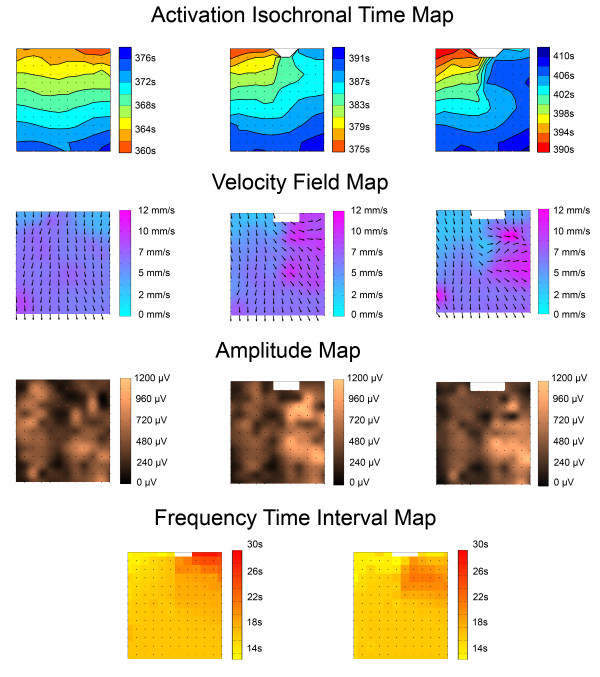
**Example Case of Gastric Dysrhythmia Mapping.** An example case of GEMS applied to mapping a gastric dysrhythmia. This high-resolution mapping experiment was performed in a weaner pig, using flexible printed circuit board electrodes [5], as per the methods described in full in [6,10]. Example activation isochronal time maps, velocity field maps, amplitude maps, and time-interval maps are shown for a sequence of 3 consecutive waves (left to right). The first wave (t = 360 s) demonstrates a normal wavefront propagating longitudinally (antegrade) (velocity 6.1 ± 1.0 mm/s; amplitude 422 ± 194 μV). The second (t = 375 s) and third (t = 390 s) waves demonstrate delayed activation of the right half of the mapped field, resulting in wavefront rotation and an abnormal area of retrograde propagation (velocity: 6.4 ± 1.9 mm/s; amplitude: 461 ± 254 μV). The mean frequency was 17 ± 2 cycles per minute. An animation of the same sequence, generated in GEMS, is shown in Figure 5.avi.

Figure5 demonstrates a specific example of the application of GEMS in the mapping of gastric dysrhythmia, in a porcine model. The GEMS outputs demonstrated in this figure include isochronal, velocity, amplitude, and time-interval maps, statistical parameters, and an animation sequence.

## Discussion

This paper presents ‘GEMS’, a new software package for the analysis and visualization of multi-electrode GI electrical recordings. This software platform effectively incorporates a number of recent analytical advances in the field of GI mapping into a coherent framework coupled to an intuitive and user-friendly GUI. The most significant value of GEMS is that it allows for substantial gains in efficiency and productivity, by automating laborious functions and quantitative analyses that must otherwise be performed manually (Table1). The package also allows for the rapid generation of high-quality graphical outputs suitable for presentation and publication.

As outlined in Table1, the software has undergone rigorous quantitative validation, with qualitative validation also achieved through application in recent experimental and clinical works [10,12]. These studies demonstrate the utility of GEMS in accelerating efforts to better understand the causes and consequences of abnormal slow wave activity occurring in disease states. Specific problems suitable to GEMS analyses include defining abnormal slow wave initiation and conduction patterns, including in clinical disorders such as gastroparesis, in which ICC numbers are reduced [10,12]. Importantly, these slow wave initiation and conduction abnormalities may often occur within normal frequency ranges, potentially being missed by techniques lacking in spatial resolution, like cutaneous EGG [10,12]. GEMS analyses have also recently been applied to show, for the first time, that high-velocity and high-amplitude activity routinely emerges in association with many dysrhythmic events [28,29]. In future, this finding may help in the localization and treatment of dysrhythmic sources.

Activation time marking, cycle clustering and isochronal mapping are all complex activities that must be undertaken with significant care to ensure accuracy is maintained and assumptions are reasonable [34]. The automated mapping algorithms currently employed in GEMS are validated and capable of producing accurate spatiotemporal maps, but must be used with caution, knowledgeable application of parameters, and in association with thorough manual review, particularly when the raw data quality is variable. To this end, GEMS is equipped with a broad range of manual analysis options.

As in cardiac mapping, some unresolved difficulties remain in the mapping process [27]. In particular, multiphasic ‘fractionated’ electrograms of long duration can occur in normal activity in the corpus [7,8], or during complex sequences, potentially introducing ambiguity into FEVT or manually-derived activation time marks [34]. Such complex activation events may arise due to electrical complexity in the propagation of wavefronts through the underlying tissue structure [35]. Currently, we adhere to a convention that the activation time of such events be marked to the first major deflection in the multiphasic deflection, and manual adjustments to FEVT results may occasionally be required. In addition, cycle clustering may be challenging when slow wave activity becomes highly disorganized, as can occur during complex dysrhythmias [9], potentially resulting in unreliable results from REGROUPS [14]. In these circumstances, as in cardiac mapping, resorting to propagation movies can be a productive solution [27], and the movie capability within GEMS is therefore a significant asset.

Another issue is that automated contour generating algorithms such as the ones employed by GEMS may incorrectly ‘assume’ that it is always permissible to interpolate between two given activation times [27]. This assumption may lead to incorrect and potentially misleading ‘crowding’ of isochrones in the presence of an activation block, which is now known to occur during a range of gastric dysrhythmias [10], requiring manual correction of the maps. Improvement on the current automated mapping algorithm to account for conduction blocks is therefore a focus of current work, as achieved previously in the cardiac field [36].

GEMS provides an extensible framework for data analysis, and we anticipate that future enhancements will continue to be added by the user community. One particular focus of interest is the application of using GEMS to analyze mapping data from other sections of the GI tract, notably the small intestine. Small intestine motility has been the focus of several HR electrical mapping studies in recent years, performed by Lammers et al. in SmoothMap [37,38]. With the recent steps toward successful human and clinical translation of HR gastric mapping [8,12], the opportunity now exists to similarly expand small intestinal mapping applications, and GEMS could be a valuable tool. However, the re-optimization of key algorithms such as FEVT to small intestinal slow waves will be necessary to ensure that accuracy and efficiency is maintained [33]. Frequency-domain analyses, such as fast-Fourier transform methods, could also be added to the software, if desired by the research community. HR electrical mapping is also now being productively applied in other excitable smooth muscle organs, such as the ureter, presenting further potential applications for GEMS [39].

To date, GEMS has only been applied to analyze GI slow wave activity. ‘Spikes’ are also described as smooth muscle action potentials, and these events have been shown to propagate in specific patterns [40]. Work is currently being undertaken to expand GEMS to allow semi-automated spike detection and mapping in the future.

Currently, GEMS is an off-line analysis system, for use after the completion of studies. Efforts are now being directed to further develop GEMS into an online mapping system suitable for real-time use [32]. In future, the software could also be adapted for analysing cutaneous signals (electrogastrography; EGG), potentially supporting the development of HR body surface EGG potential mapping, as proposed recently by Du et al. [41]. However, the algorithms are presently designed for serosal data and would therefore need extensive modifications to be useful for EGG studies.

## Conclusions

This work has introduced a new efficient and intuitive software package, GEMS (Gastrointestinal Electrical Mapping Suite), for analyzing and visualizing high-resolution multi-electrode gastrointestinal mapping data. The software is designed to be used by non-experts in data and signal processing, and is intended to be used by clinical researchers as well as physiologists and bioengineers. The use and distribution of this package will greatly accelerate efforts to improve the understanding of the causes and clinical significance of gastrointestinal electrical disorders through high-resolution mapping.

## Availability and requirements

The GEMS software is currently available for academic use (under copyright), via the project website: *http://sites.google.com/site/gimappingsuite*. The project website also includes a bibliography of papers relevant to GEMS, updates and development notes, a list of contributors, and a feedback system for the user community to request bug fixes and/or new features. The current version at time of writing is GEMS v1.4. Update releases are routinely notified on the project website.

GEMS has been developed in MATLAB R2009a. To run GEMS.exe it is required to have Matlab Compiler Runtime (MCR), which can be obtained with GEMS.exe at *http://sites.google.com/site/gimappingsuite*. Users currently require a PC computer with Windows. Linux and Mac OS X versions could also be made available if desired by the user community.

A step-by-step user manual has been written to guide GEMS use, written in lay language to accommodate users who are unfamiliar with the technical details of signal processing and programming (available as additional file 2 with this paper). The manual includes detailed illustrations of the GEMS interface, and explains the numerous functions and options that are available. The latest user manual can be downloaded from the project website above, and can also be accessed by the user from within GEMS at any time during the processing of data.

## Abbreviations

FEVT, Falling-edge variable threshold; GEMS, Gastrointestinal electrical mapping suite; GI, Gastrointestinal; GUI, Graphical user interface; HR, High-resolution; ICC, Interstitial cells of Cajal; REGROUPS, Region-growing using polynomial surface estimate stablization; SIV, Spatial interpolation and visualization.

## Competing interests

Authors GOG, NP, PD, TRA, AJP, LKC and JE hold intellectual property in the field of GI multi-electrode mapping. Patented material includes algorithms used to automatically process extracellular slow wave signals. However, this software is being freely distributed for non-commercial use by academics, and no authors have any financial interests whatsoever in relation to the publication of the work. No commercial funding has been used in the work, and no reimbursements, fees, funding or salary are being sought or given in relation to its use as specified in this manuscript.

## Authors’ contributions

**RY:** Project management, software design and coding, drafting of manuscript. **GOG**: software design, validation and testing, drafting of manuscript. **NP,PD**: software design and coding, critical revision of manuscript. **TRA**: software design and testing, critical revision of manuscript. **AJP, LKC**: supervision, critical revision of manuscript; **JCE**: project founder, software design and coding, critical revision of manuscript. All authors read and approved the final manuscript.

## Important note on conditions of use

Users must note that GEMS use is governed by the terms and conditions provided in the document provided (‘GEMS_EndUserLicenseAgreement.docx’). Academic users may freely use the software for research purposes, but cannot use it for clinical or commercial purposes, and cannot redistribute the software. There are no warranties, and the authors and their employees will not be held liable for any damages arising in any way out of the use of GEMS, including through its application to patient care.

## Supplementary Material

Additional file 1 Fig5.avi presents an example of a slow wave propagation animation generated using GEMS, and is available as an additional file (refer Fig. 5 caption for further information). Click here for file

Additional file 2 **The GEMS user manual has been uploaded as an additional file.** The latest version of GEMS, the matlab runtime application, the user manual, project updates, support, and documentation can be found (free to academics without restriction) on the project website: *http://sites.google.com/site/gimappingsuite*. Please be patient while the software opens at its first use. Click here for file

## References

[B1] Huizinga JD Lammers WJEP Gut peristalsis is coordinated by a multitude of cooperating mechanisms Am J Physiol Gastrointest Liver Physiol 2009 296 1 8 10.1152/ajpgi.90380.2008 18988693

[B2] Chen JD Lin Z Yin Y Parkman HP, McCallum RW, Rao SC Electrogastrography In GI Motility Testing: A Laboratory and Office Handbook 2011 SLACK Incorporated, Thorofare 81 92

[B3] Leahy A Besherdas K Clayman C Mason I Epstein O Abnormalities of the electrogastrogram in functional gastrointestinal disorders Am J Gastroenterol 1999 94 1023 1028 10201477 10.1111/j.1572-0241.1999.01007.x

[B4] Grover M Farrugia G Lurken MS Cellular changes in diabetic and idiopathic gastroparesis Gastroenterology 2011 140 1575 1585 e8 21300066 10.1053/j.gastro.2011.01.046 PMC3081914

[B5] Du P O'Grady G Egbuji JU High-resolution mapping of in vivo gastrointestinal slow wave activity using flexible printed circuit board electrodes: methodology and validation Ann Biomed Eng 2009 37 839 846 19224368 10.1007/s10439-009-9654-9 PMC4090363

[B6] Egbuji JU O'Grady G Du P Origin, propagation and regional characteristics of porcine gastric slow wave activity determined by high-resolution mapping Neurogastroenterol Motil 2010 22 e292 e300 20618830 10.1111/j.1365-2982.2010.01538.x PMC4110485

[B7] Lammers WJ Ver Donck L Stephen B Smets D Schuurkes JA Origin and propagation of the slow wave in the canine stomach: the outlines of a gastric conduction system Am J Physiol Gastrointest Liver Physiol 2009 296 1200 1210 10.1152/ajpgi.90581.2008 19359425

[B8] O'Grady G Du P Cheng LK The origin and propagation of human gastric slow wave activity defined by high-resolution mapping Am J Physiol Gastrointest Liver Physiol 2010 299 585 592 10.1152/ajpgi.00125.2010 PMC2950696 20595620

[B9] Lammers WJEP Ver Donck L Stephen B Smets D Schuurkes JAJ Focal activities and re-entrant propagations as mechanisms of gastric tachyarrhythmias Gastroenterology 2008 135 1601 1611 18713627 10.1053/j.gastro.2008.07.020

[B10] O'Grady G Egbuji JU Du P High-resolution spatial analysis of slow wave initiation and conduction in porcine gastric dysrhythmia Neurogastroenterol Motil 2011 23 e345 e355 21714831 10.1111/j.1365-2982.2011.01739.x PMC3156377

[B11] O'Grady G Du P Lammers WJ High-resolution entrainment mapping for gastric pacing: a new analytic tool Am J Physiol Gastrointest Liver Physiol 2010 298 314 321 10.1152/ajpgi.00389.2009 PMC2822498 19926815

[B12] O'Grady G Angeli TR Cheng LK Aberrant initiation and conduction of slow wave activity in diabetic and idiopathic gastroparesis Gastroenterology 2011 140 S705 S706

[B13] Erickson JC O'Grady G Du P Falling-edge, variable threshold (FEVT) method for the automated detection of gastric slow wave events in serosal high-resolution electrical recordings Ann Biomed Eng 2010 38 1511 1529 20024624 10.1007/s10439-009-9870-3 PMC2855965

[B14] Erickson JC O'Grady G Du P Egbuji JU Pullan AJ Cheng LK Automated cycle partitioning and visualization of high-resolution activation time maps of gastric slow wave recordings: the Region Growing Using Polynomial Surface-estimate stabilization (REGROUPS) Algorithm Ann Biomed Eng 2011 39 469 483 20927594 10.1007/s10439-010-0170-8 PMC4127312

[B15] Paskaranandavadivel N O'Grady G Du P Pullan AJ Cheng LK An improved method for the estimation and visualization of velocity fields from gastric high-resolution electrical mapping. IEEE Trans Biomed Eng 2012 10.1109/TBME.2011.2181845 PMC4106919 22207635

[B16] Ideker RE Smith WM Wolf P Danieley ND Bartram FR Simultaneous multichannel cardiac mapping systems Pacing Clin Electrophysiol 1987 10 281 292 2437532 10.1111/j.1540-8159.1987.tb05966.x

[B17] Rogers JM Bayly PV Ideker RE Smith WM Quantitative techniques for analyzing high-resolution cardiac-mapping data IEEE Eng Med Biol Mag 1998 17 62 72 9460622 10.1109/51.646223

[B18] Potse M Linnenbank AC Grimbergen CA Software design for analysis of multichannel intracardial and body surface electrocardiograms Comp Meth Prog Biomed 2002 69 225 236 10.1016/s0169-2607(02)00014-7 12204450

[B19] Shenasa M Hindricks G Borggrefe M Breithardt G Cardiac Mapping 3rd Edition 2009 Blackwell Publishing Ltd, Oxford

[B20] Lammers WJ SmoothMap [Computer Program]. Version 3.05. Al Ain, United Arab Emirates 2009 http://www.smoothmap.org

[B21] Paskaranandavadivel N Cheng LK Du P O'Grady G Pullan AJ Improved signal processing techniques for the analysis of high resolution serosal slow wave activity in the stomach Conf Proc IEEE Eng Med Biol Soc 2011 1737 1740 22254662 10.1109/IEMBS.2011.6090497 PMC4071157

[B22] O'Grady G Gastrointestinal extracellular electrical recordings: fact or artifact? Neurogastroenterol Motil 2012 24 1 6 22188324 10.1111/j.1365-2982.2011.01815.x PMC3245636

[B23] Zhang D Wavelet approach for ECG baseline wander correction and noise reduction Conf Proc IEEE Eng Med Biol Sci 2005 1212 1215 10.1109/IEMBS.2005.1616642 17282411

[B24] Savitzky A Golay MJE Smoothing and differentiation of data by simplified least squares procedures Anal Chem 1964 36 1627 1639

[B25] Park SB Noh YS Park SJ Yoon HR An improved algorithm for respiration signal extraction from electrocardiogram measured by conductive textile electrodes using instantaneous frequency estimation Med Biol Eng Comput 2008 46 147 158 18210178 10.1007/s11517-007-0302-y PMC2668578

[B26] O'Grady G Paskaranandavadivel N Angeli T A comparison of gold vs silver electrode contacts for high-resolution gastric electrical mapping using flexible printed circuit board electrodes Physiol Meas 2011 32 N13 N22 21252419 10.1088/0967-3334/32/3/N02 PMC4127313

[B27] Rogers JM Bayly PV Cabo C, Rosenbaum DS Quantitative Analysis of Complex Rhythms In Quantitative Cardiac Electrophysiology 2002 Marcel Decker Inc, New York 403 428

[B28] O'Grady G Angeli TR Cheng LK Emergence of circumferential slow wave propagation during gastric dysrhythmias in diabetic gastroparesis Gastroenterology 2011 140 705

[B29] Du P O'Grady G Paskaranandavadivel N Quantification of velocity anisotropy during gastric electrical arrhythmia Conf Proc IEEE Eng Med Biol Soc 2011 4402 4405 22255315 10.1109/IEMBS.2011.6091092 PMC4127317

[B30] Du P Qiao W O'Grady G Automated detection of gastric slow wave events and estimation of propagation velocity vector fields from serosal high-resolution mapping Conf Proc IEEE Eng Med Biol Sci 2009 2527 2530 10.1109/IEMBS.2009.5334822 PMC4081486 19964973

[B31] Hazewinkel M Point of inflection 2001 Springer, New York

[B32] Bull S O'Grady G Cheng LK Pullan AJ A framework for the online analysis of multi-electrode gastric slow wave recordings Conf Proc IEEE Eng Med Biol Soc 2011 1741 1744 22254663 10.1109/IEMBS.2011.6090498 PMC4108589

[B33] Angeli TR O'Grady G Erickson JC Mapping small intestine bioelectrical activity using high-resolution printed-circuit-board electrodes Conf Proc IEEE Eng Med Biol Soc 2011 4951 4954 22255449 10.1109/IEMBS.2011.6091227 PMC4076342

[B34] Ideker RE Smith WM Blanchard SM The assumptions of isochronal cardiac mapping Pacing Clin Electrophysiol 1989 12 456 478 2466272 10.1111/j.1540-8159.1989.tb02684.x

[B35] de Bakker JM Wittkampf FH The pathophysiologic basis of fractionated and complex electrograms and the impact of recording techniques on their detection and interpretation Circ Arrhythm Electrophysiol 2010 3 204 213 20407105 10.1161/CIRCEP.109.904763

[B36] Potse M Linnenbank AC Grimbergen CA Automated generation of isochronal maps in the presence of activation block Int J Bioelectromagnetism 2002 4 115 116

[B37] Lammers WJ Ver Donck L Schuurkes JA Stephen B Peripheral pacemakers and patterns of slow wave propagation in the canine small intestine in vivo Can J Physiol Pharmacol 2005 83 1031 1043 16391712 10.1139/y05-084

[B38] Lammers WJ Stephen B Slack JR Dhanasekaran S Anisotropic propagation in the small intestine Neurogastroenterol Motil 2002 14 357 364 12213103 10.1046/j.1365-2982.2002.00340.x

[B39] Hammad FT Lammers WJ Stephen B Lubbad L Propagation characteristics of the electrical impulse in the normal and obstructed ureter as determined at high electrophysiological resolution BJU Int 2011 108 E36 E42 21044242 10.1111/j.1464-410X.2010.09793.x

[B40] Lammers WJ Donck LV Schuurkes JA Stephen B Longitudinal and circumferential spike patches in the canine small intestine in vivo Am J Physiol Gastrointest Liver Physiol 2003 285 G1014 G1027 12842824 10.1152/ajpgi.00138.2003

[B41] Du P O'Grady G Cheng LK Pullan AJ A multi-scale model of the electrophysiological basis of the human electrogastrogram Biophys J 2010 99 2784 2792 21044575 10.1016/j.bpj.2010.08.067 PMC2965998

