# Supplementary material for: The gastrointestinal electrical mapping suite (GEMS): software for analyzing and visualizing high-resolution (multi-electrode) recordings in spatiotemporal detail
Source: BMC Gastroenterol. 2012 Jun 6;12:60. doi: 10.1186/1471-230X-12-60 (PMC3464652; doi:10.1186/1471-230X-12-60)
Supplement: Additional file 2 — The GEMS user manual has been uploaded as an additional file. The latest version of GEMS, the matlab runtime application, the user manual, project updates, support, and documentation can be found (free to academics without restriction) on the project website: http://sites.google.com/site/gimappingsuite. Please be patient while the software opens at its first use. [file 1471-230X-12-60-S2.pdf]

2011

User Manual

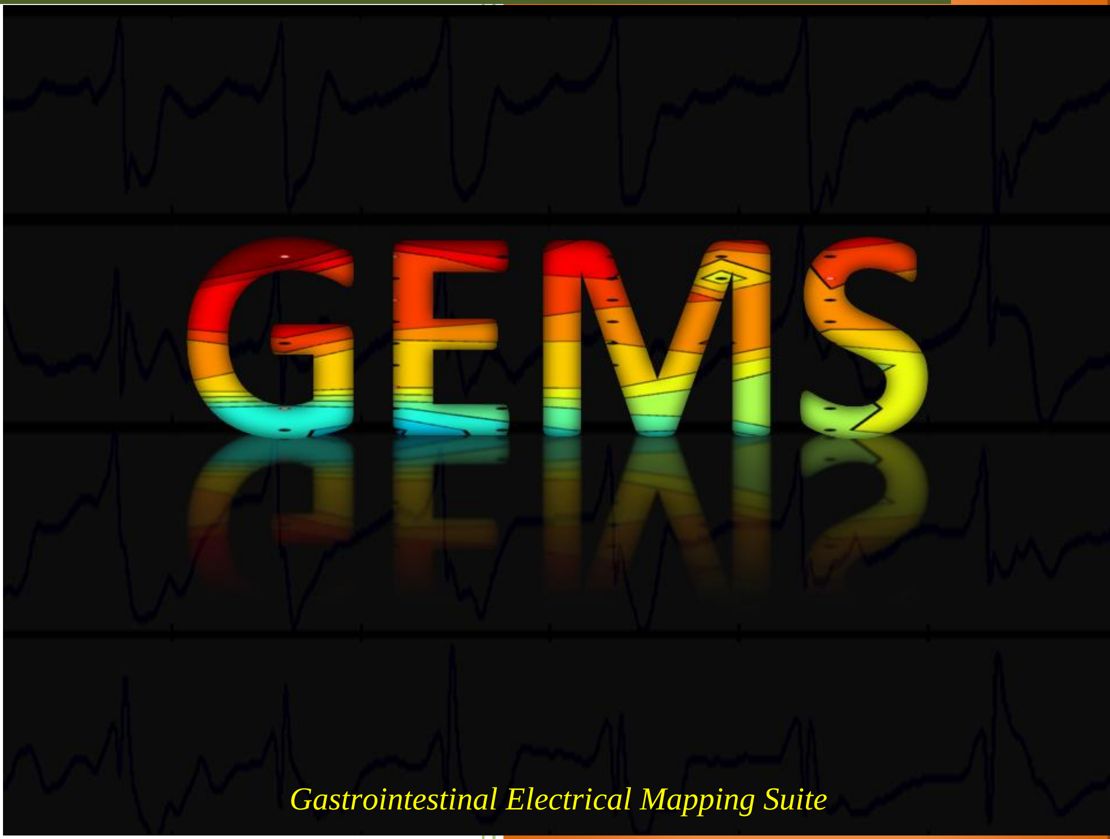

Auckland UniServices Limited

and

Jonathan C Erickson

Developers: Jonathan C Erickson
Rita Yassi
Nira Paskaranandavadivel

Contact us: gems.academic@gmail.com

Website: ***https://sites.google.com/site/gimappingsuite/***

Written by: Rita Yassi
Jonathan C Erickson

Table of Contents

1 Software overview 1
2 System requirements 1
2.1 Binary 1
2.2 Open Source 1
3 Installation 1
3.1 Binary 1
3.2 Open Source 2
4 Files required for GEMS 2
4.1 Raw recorded data file 2
4.2 Electrode configuration file 4
5 GEMS architecture 5
6 Launching 7
6.1 Binary 7
6.2 Open Source 7
7 Electrode configuration file 10
8 Default parameters 12
9 Main GEMS figure controls 14
10 Pre-screen 16
11 Auto-marking 18
12 Post-processing 23
13 Time/Amplitude/Velocity/Interval/Downstroke width maps 27
14 Flashlight movie 29
15 Statistical analysis 31
16 Saving data 32
17 To re-load analysed data 32
18 Useful Hints 33
19 Glossary of parameters 34
20 For developers (open source only) 38
21 Back-end information 40
21.1 Clustering algorithm 40
21.2 FEVT detection 40

22 References 41
23 Contributors 43

1 Software overview

This user friendly GUI module is intended to facilitate (and accelerate) electrode data analysis to quickly visualize and quantify a series of activation maps and velocity fields. It incorporates algorithm for automatically detecting activation times, and partitioning each individual slow wave event so that isochronal maps and velocity field maps can also be generated with minimal manual tedious labour.

This manual will help guide you through the usage of the module, point out some of its features, and illustrate its capabilities. Any comments or questions should be addressed to GEMS management team by email: gems.academic@gmail.com.

2 System requirements

Operating Systems

- Windows 7
- Windows XP

2.1 Binary

To use GEMS, you will need the Matlab Complier Runtime (MCR) installed on your machine. The MCR will be supplied with GEMS.exe.

2.2 Open Source

To use GEMS, you will need the following installed on your machine:

1. MATLAB® R2009a or above. For more information on how you purchase and install MATLAB® please visit Mathworks.
2. The wavelet toolbox is needed for some baseline drift and filtering methods.
3. Winrar or equivalent. To download winrar for free visit Winrar.
4. For developers, perl is required for some functionalities to work. For window users, to download perl for free visit ActivePerl.

3 Installation

3.1 Binary

1. Download GEMS.zip and save it on your computer.
2. Extract the zip file. You will have GEMS_pkg.exe, License folder and exampleFiles folder.
3. Double click on GEMS_pkg.exe, this will prompt you to install MCR. Follow the prompts.
4. To launch GEMS go to Section 6.1.

3.2 Open Source

1. Create a GEMS folder in your machine.
2. Download GEMS.rar and save it inside the GEMS folder.
3. Extract all the files inside GEMS.rar. If you have winrar installed, this usually can be done by right clicking on GEMS.rar, and then selecting **Extract Here**.
4. When extraction is finished, you should be able to see a set of folders as shown in Figure 1.
5. To launch go to Section 6.2.

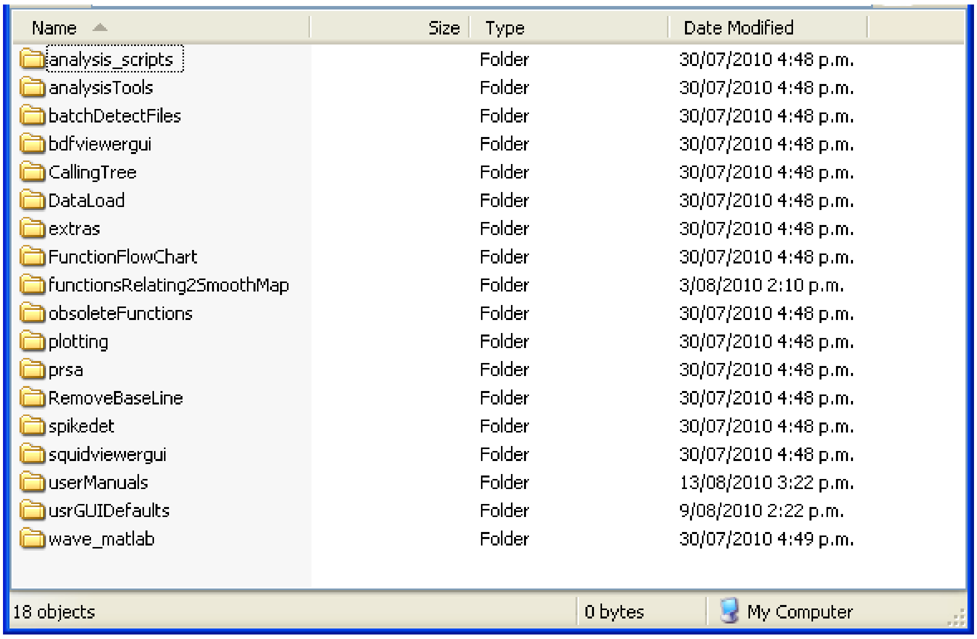

Name	Size	Type	Date Modified
analysis_scripts		Folder	30/07/2010 4:48 p.m.
analysisTools		Folder	30/07/2010 4:48 p.m.
batchDetectFiles		Folder	30/07/2010 4:48 p.m.
bdfviewergui		Folder	30/07/2010 4:48 p.m.
CallingTree		Folder	30/07/2010 4:48 p.m.
DataLoad		Folder	30/07/2010 4:48 p.m.
extras		Folder	30/07/2010 4:48 p.m.
FunctionFlowChart		Folder	30/07/2010 4:48 p.m.
functionsRelating2SmoothMap		Folder	3/08/2010 2:10 p.m.
obsoleteFunctions		Folder	30/07/2010 4:48 p.m.
plotting		Folder	30/07/2010 4:48 p.m.
prsa		Folder	30/07/2010 4:48 p.m.
RemoveBaseLine		Folder	30/07/2010 4:48 p.m.
spikedet		Folder	30/07/2010 4:48 p.m.
squidviewergui		Folder	30/07/2010 4:48 p.m.
userManuals		Folder	13/08/2010 3:22 p.m.
usrGUIDefaults		Folder	9/08/2010 2:22 p.m.
wave_matlab		Folder	30/07/2010 4:49 p.m.

18 objects | 0 bytes | My Computer

Figure 1: Extracted GEMS folders.

4 Files required for GEMS

The only file needed for GEMS to work is a file that contains the raw recorded data. Other files can also be read into GEMS which contain extra information.

4.1 Raw recorded data file

There are 3 types of files that can be important into GEMS

1. A '.bdf' file usually obtained from the Biosemi system.
2. A '.mat' file with the following format: any file containing stored variables named "ElecData", "resamp", and "fs" can be used. ElecData is an [Nsigs x Nsamps] data matrix (data in rows); resamp is a Boolean (0/1) specifying whether or not the data has been/is to be resampled prior to loading; fs is a scalar specified the original sampling frequency.
3. '.txt' files:
    a. A '.txt' file from Unemap with the following format: first line starting with the word *time* followed by the number of channels or electrodes. The first column contains the times in seconds, the potentials for each time and electrode are saved in the remaining columns as shown in Figure 2.

time	1	2	3	4	5	6	7	8	9	10	11	12	13	14	15	16	17	18	19	20	21	22	23	24	25	26	27
2.00000000	-1.07479	-0.159529	-0.95501	-0.93989	0.0159975	-0.120135	-1.83425	-0.202355	-0.617954	0.05970
4.00000000	-1.13595	-0.180106	-1.03746	-0.832639	-0.00199575	-0.149172	-1.93201	-0.232817	-0.517243	0.0
6.00000000	-1.11128	-0.154438	-1.1369	-0.781821	-0.0312959	-0.186858	-2.09367	-0.280168	-0.339105	0.0
8.00000000	-1.09522	-0.131208	-1.21388	-0.853866	-0.0496652	-0.215207	-2.23064	-0.321178	-0.260805	0.0
10.00000000	-1.0472	-0.0943966	-1.26633	-1.0235	-0.063171	-0.237376	-2.34353	-0.359582	-0.225971	-0.
12.00000000	-1.00008	-0.0516211	-1.32653	-1.21191	-0.0750356	-0.257378	-2.48516	-0.397027	-0.197661	-0.
14.00000000	-0.949082	-0.00872719	-1.37917	-1.38196	-0.0858727	-0.27465	-2.61444	-0.430274	-0.165955	-0.
16.00000000	-0.797723	0.0824068	-1.39393	-1.51487	-0.0939782	-0.272889	-2.73951	-0.442154	-0.0748192
18.00000000	-0.650121	0.168763	-1.39014	-1.68351	-0.0968432	-0.265959	-2.83148	-0.448643	-0.0245979	-0.
20.00000000	-0.585999	0.205905	-1.38369	-1.82843	-0.0961844	-0.264009	-2.86189	-0.455793	-0.0384216	-0.
22.00000000	-0.559394	0.221617	-1.3643	-1.87884	-0.0897977	-0.253311	-2.85806	-0.446237	-0.0659412	-0.
24.00000000	-0.516607	0.242808	-1.31605	-1.99629	-0.0758389	-0.232362	-2.8211	-0.426965	-0.149904	-0.
26.00000000	-0.429183	0.300994	-1.31305	-2.1664	-0.0718164	-0.221577	-2.90178	-0.42634	-0.174811	-0.04045
28.00000000	-0.428969	0.288155	-1.2449	-2.1421	-0.0562132	-0.197511	-2.78789	-0.393578	-0.250334	-0.
30.00000000	-0.440615	0.273251	-1.20124	-2.09423	-0.0458262	-0.181354	-2.7089	-0.36972	-0.296663	-0.01906
32.00000000	-0.503196	0.217784	-1.09705	-1.92161	-0.0200037	-0.142965	-2.49868	-0.308742	-0.408725	0.0
34.00000000	-0.540529	0.168116	-0.987188	-1.73657	0.000857842	-0.109288	-2.26861	-0.253118	-0.486594
36.00000000	-0.653668	0.0601639	-0.797626	-1.41673	0.0427223	-0.0481075	-1.85941	-0.151636	-0.67121
38.00000000	-0.785326	-0.00736415	-0.847687	-1.28421	0.0349196	-0.0706	-1.85541	-0.168375	-0.661282
40.00000000	-0.923239	-0.07951	-0.898081	-1.12534	0.0264826	-0.0935275	-1.84591	-0.184011	-0.645604
42.00000000	-1.04907	-0.141513	-0.975353	-0.976389	0.0100206	-0.127455	-1.88207	-0.214788	-0.58439
44.00000000	-1.12401	-0.172818	-1.04432	-0.844226	-0.00457327	-0.152018	-1.94849	-0.237476	-0.502435
46.00000000	-1.11181	-0.152578	-1.14699	-0.780125	-0.0334209	-0.189889	-2.11142	-0.284153	-0.327165
48.00000000	-1.10904	-0.141171	-1.20097	-0.838392	-0.0459153	-0.210696	-2.20174	-0.313913	-0.282592
50.00000000	-1.05698	-0.0936149	-1.28637	-1.00497	-0.0657841	-0.241025	-2.38057	-0.363778	-0.206025	-0.
52.00000000	-0.979175	-0.0397615	-1.33351	-1.21568	-0.0784937	-0.260039	-2.50572	-0.401523	-0.170107
54.00000000	-0.961727	-0.0164411	-1.37529	-1.37161	-0.0843062	-0.273367	-2.60137	-0.427477	-0.17863	-0.
56.00000000	-0.806966	0.0782322	-1.39964	-1.51298	-0.0948919	-0.275368	-2.74317	-0.444838	-0.0745424
58.00000000	-0.651324	0.168068	-1.40475	-1.68494	-0.102045	-0.274295	-2.84541	-0.458948	-0.00244289	-0.
60.00000000	-0.616013	0.188889	-1.39188	-1.77196	-0.0980636	-0.268074	-2.85299	-0.456937	-0.027451	-0.

Figure 2: Unemap '.txt' file containing raw recorded data.

b. A ‘.txt’ file from SmoothMap with the following format: the first entry is the total number of header rows, second entry is the number of electrodes, third entry is the total number of rows of entry, fourth entry is the frequency in Hz, the following 6 rows are just 1s as shown in Figure 3. The data is listed is so that each row represents data for all electrodes per time t.

10
160
57984
128
1
1
1
1
1
1
4.507	2.617	4.604	4.002	4.281	4.180	3.570	4.246	4.128	4.208	4.251	3.277	3.786	4.501
4.608	2.700	4.704	4.096	4.385	4.280	3.648	4.351	4.222	4.302	4.337	3.365	3.878	4.601
4.709	2.784	4.804	4.189	4.489	4.379	3.726	4.456	4.317	4.396	4.423	3.453	3.970	4.700
4.809	2.867	4.903	4.281	4.592	4.478	3.803	4.560	4.411	4.490	4.509	3.540	4.062	4.799
4.909	2.950	5.001	4.373	4.694	4.575	3.880	4.664	4.504	4.583	4.593	3.627	4.153	4.897
5.008	3.032	5.099	4.464	4.796	4.673	3.956	4.767	4.597	4.676	4.678	3.713	4.243	4.994
5.106	3.113	5.196	4.555	4.897	4.770	4.032	4.869	4.689	4.768	4.762	3.798	4.333	5.091
5.204	3.194	5.293	4.645	4.998	4.866	4.108	4.971	4.780	4.859	4.845	3.883	4.423	5.187
5.301	3.275	5.389	4.735	5.098	4.961	4.183	5.072	4.871	4.950	4.928	3.968	4.512	5.283
5.398	3.355	5.485	4.824	5.197	5.057	4.257	5.173	4.962	5.040	5.010	4.052	4.600	5.378
5.494	3.435	5.580	4.912	5.296	5.151	4.332	5.273	5.052	5.130	5.092	4.136	4.688	5.473
5.590	3.514	5.674	5.000	5.395	5.245	4.405	5.372	5.141	5.219	5.174	4.219	4.775	5.567
5.685	3.593	5.768	5.088	5.492	5.339	4.479	5.471	5.230	5.308	5.255	4.302	4.862	5.661
5.780	3.672	5.861	5.175	5.589	5.432	4.552	5.569	5.318	5.396	5.335	4.384	4.948	5.754
5.873	3.750	5.954	5.261	5.686	5.524	4.624	5.667	5.406	5.484	5.415	4.466	5.034	5.846
5.967	3.827	6.046	5.347	5.782	5.616	4.696	5.764	5.493	5.571	5.494	4.547	5.119	5.938
6.060	3.904	6.138	5.433	5.878	5.707	4.767	5.860	5.580	5.658	5.573	4.628	5.204	6.029
6.152	3.981	6.229	5.518	5.972	5.797	4.839	5.956	5.667	5.744	5.652	4.708	5.289	6.120
6.243	4.057	6.319	5.602	6.067	5.888	4.909	6.052	5.752	5.829	5.730	4.788	5.372	6.210
6.334	4.133	6.409	5.686	6.160	5.977	4.979	6.146	5.837	5.914	5.807	4.867	5.456	6.300
6.425	4.208	6.499	5.770	6.253	6.066	5.049	6.240	5.922	5.999	5.884	4.946	5.538	6.389

Figure 3: SmoothMap '.txt' file containing raw recorded data.

c. A simple ‘.txt’ file with the same format as the SmoothMap ‘.txt’ file format (as shown in Figure 3) with no headers.

4.2 Electrode configuration file

A '.txt' file, usually referred to as electrode configuration or matrix file. The file contains the list of electrodes and their orientation as shown in Figure 4. This file can be generated manually or using GEMS (outlined further in Section 7).

The first line of the file is a default setup line, just set it as shown in Figure 4.

The first entry of the second line is the total number of channels recorded from (160 in Figure 4).

The second entry of the second line is the number of columns (8 in Figure 4).

The third entry of the second line is the number of rows (20 Figure 4).

The final entry of the second line is the inter-electrode spacing in 'mm' (7.62 mm in Figure 4).

Third line should be empty.

Fourth line onwards contains the electrode configuration (electrode numbers) used in the experiment.

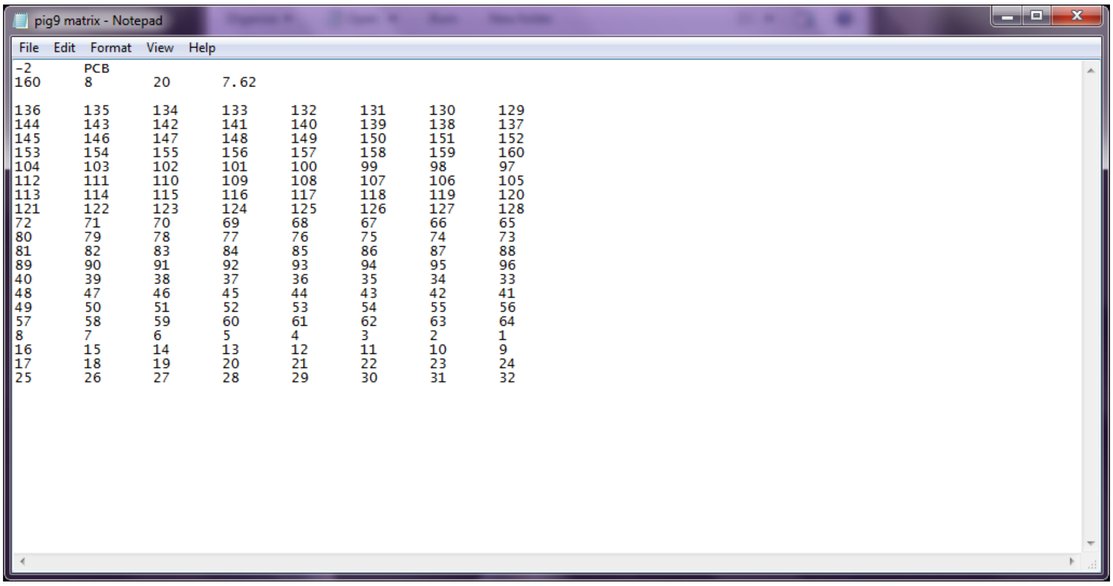


```
-2	PCB
160	8	20	7.62

136	135	134	133	132	131	130	129
144	143	142	141	140	139	138	137
145	146	147	148	149	150	151	152
153	154	155	156	157	158	159	160
104	103	102	101	100	99	98	97
112	111	110	109	108	107	106	105
113	114	115	116	117	118	119	120
121	122	123	124	125	126	127	128
72	71	70	69	68	67	66	65
80	79	78	77	76	75	74	73
81	82	83	84	85	86	87	88
89	90	91	92	93	94	95	96
40	39	38	37	36	35	34	33
48	47	46	45	44	43	42	41
49	50	51	52	53	54	55	56
57	58	59	60	61	62	63	64
8	7	6	5	4	3	2	1
16	15	14	13	12	11	10	9
17	18	19	20	21	22	23	24
25	26	27	28	29	30	31	32
```

Figure 4: An example of electrode configuration file.

5 GEMS architecture

Data analysis in GEMS is divided into three stages: pre-processing, processing and post-processing as shown in Figure 5. In the pre-processing stage, the ‘raw recorded data’ input is converted to a file that is visualized and filtered in MATLAB. Channel/electrode selection controls allow the user to discard electrodes with no reliable recorded data (e.g., due to poor contact of the electrode with the GI tract or a technical fault). The output of the pre-processing stage is the ‘filtered data’ which becomes the input for the processing stage. Using the in-built algorithms, activation times can be automatically detected and the marked events can be partitioned and grouped/clustered into a series of wave-fronts. The output of the processing stage is ‘marked clustered events’, which becomes the input for the final post-processing stage. In this stage, pseudo-colored contour maps can be produced to show the propagation and distribution of activation times, amplitudes and velocity fields. In addition, propagation movies can be produced to allow animated visualization of the spread of electrical activity.

At each stage, the user can interact with the program, perform processing steps, and tune multiple parameters to their needs via the user-friendly GUI applications. Further explanations of each stage are provided below.

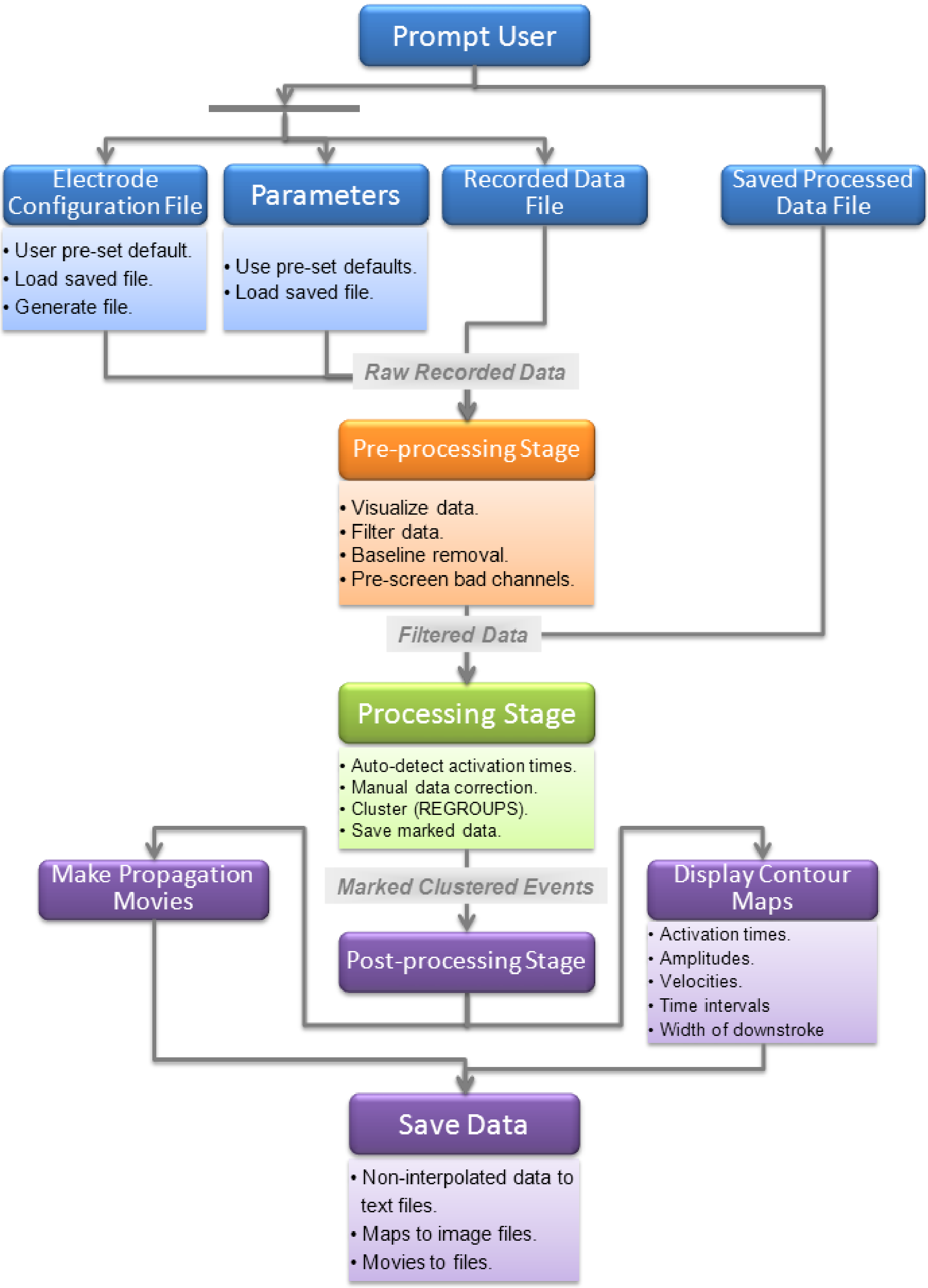

Figure 5: GEMS flow-chart.

6 Launching

6.1 Binary

Double click GEMS.exe and move to step 1.

6.2 Open Source

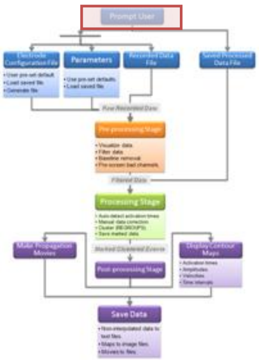

a) Launch MATLAB® either by double clicking on the icon on your desktop or by using Start-Programs-Matlab or by locating it on your machine.

b) Add GEMS directory and subdirectories by typing in the MATLAB command window **addpath(genpath('path/directory'))** (see Figure 6).

   a. for example: addpath(genpath('d:\Users\rita\GEMS'))

c) Launch GEMS by typing in MATLAB command window **GEMS** as shown in Figure 6.

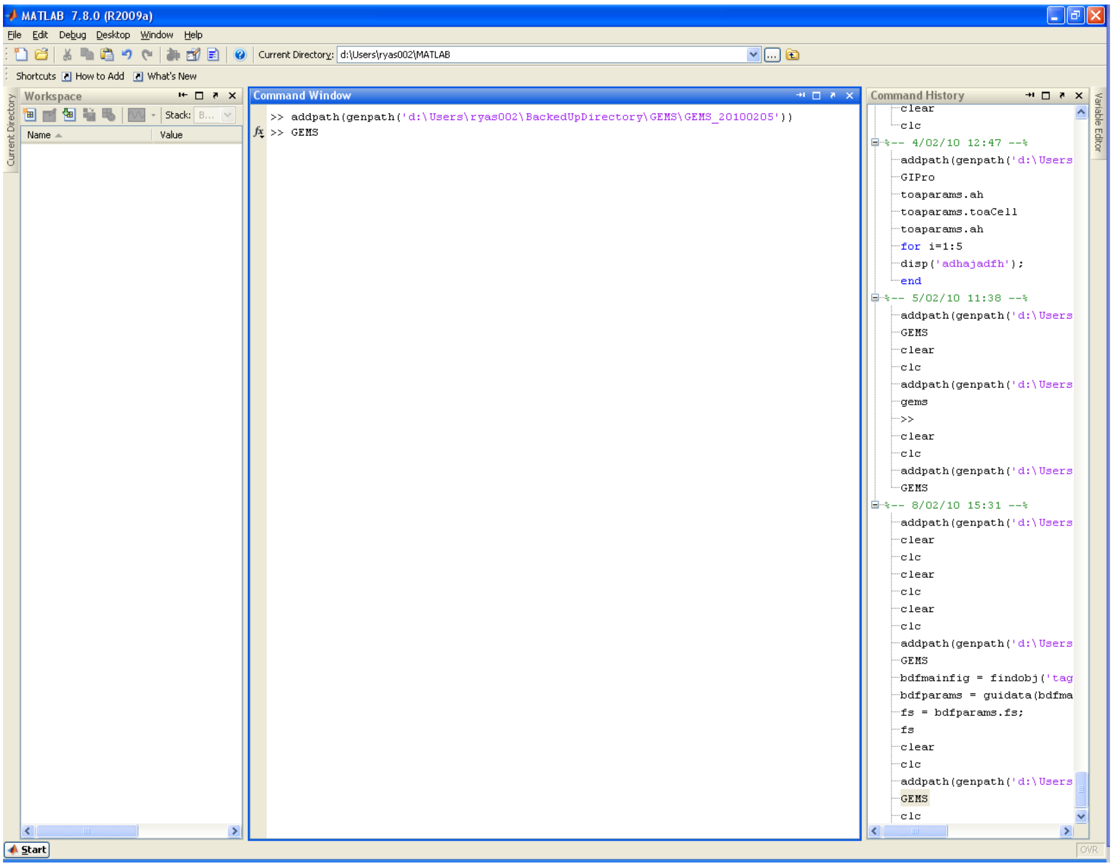

Figure 6: Launching GEMS in MATLAB command window.

1. A Start-up figure will pop up as shown in Figure 7. The boxes show the default files. To change them click on the open file icon next to each entry. The left hand side of the figure is associated with raw files. On the left hand side, the first box is the main '.bdf', '.mat' or '.txt' file which contains the main raw recorded data (refer to Section 4).
2. The second box is for the parameters. Users have 3 options;

a. Use the default parameters saved in the defaultParameters.m function file.
b. Set parameters by clicking on the *Set Parameters* button, this will open up the parameter figure and you can set the parameters as desired. Remember to click on *Change Parameters* then press *Close Me*. Never close this figure using the close button 'X'.
c. Load in a '.mat' file, this is usually available if you have used GEMS before and you saved it.

3. The third entry is the electrode configuration file. If not entered GEMS will read some default file it has. The fourth entry is the channel number to show when the main GEMS figure is launched.
4. The box on the right hand side is related to '.mat' files which have analysed data. When user uses this, there will be no need to set the other files on the left hand side of the figure, everything is saved in the analysed data file. If user opens analysed file, then you can skip to Section 11.
NOTES:
- After the first time you read '.bdf' or '.txt' file which contains the raw data, a '.mat' file will be generated, so from then on you can use the '.mat' file as it will be faster to read.
- At load time, GEMS checks to see if the parameter 'fs' in the '.mat' file matches the requested (re)sampling frequency in the default '.m' file. If these two values do not match, GEMS attempt to reload the data from the original '.bdf' or '.txt' file, resampling at the rate specified in the default '.m' file. The practical consequence is that if you are creating a '.mat' file from scratch you must do two things: 1) resample your data prior to saving it in the '.mat' file and 2) make sure the frequency saved in your '.mat' file (field 'fs') matches the value stored in default '.m' file. Otherwise, an error will be thrown and your data will not load.

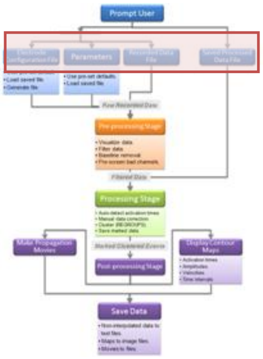

5. If user wants to process an entire file (all channels and all time) check the *Process entire file* checkbox in Figure 7.
6. Once finished, click on *Done* and a GEMS figure will pop-up (see Figure 8) which shows the first 120 seconds of the first 10 channels (this is the default setup).
NOTES:
- If you are reloading marked files from this step, the main GEMS figure will not be generated.
- If the analysed data was saved using an old version of GEMS, user might be prompted to read in the original '.bdf/.mat' file.

NOTE:

GEMS has default files that can be used to run GEMS. In the open source the files are pre-set in the boxes. User can press the *Done* button and the main GEMS figure should pop-up. For the binary, the user can locate the example files in the exampleFiles folder. The included files are as follows:

- Raw data files: ExampleFile_rawdata_pig10exp2_0To300.mat and ExampleFile_rawdata_pig10exp2_0To100.mat. These can be read in the "Read in the main bdf/txt/mat file" box.
- Parameter file: ExampleFile_pig10exp2_0To100_defaultParameters.mat, this can be read in the "Set parameters or Read in parameter" box.

- Electrode configuration file: ExampleFile_elecConfig_pig10.txt, this can be read into the “Read in the electrode config file” box.
- Marked file: ExampleFile_markeddata_pig10exp2_0To300.mat, this can be read into the “Read in the analysed mat file” box. Once user presses *Done*, GEMS will launch the AT figure containing marked data. If user opens analysed file, then you can skip to Section 11.

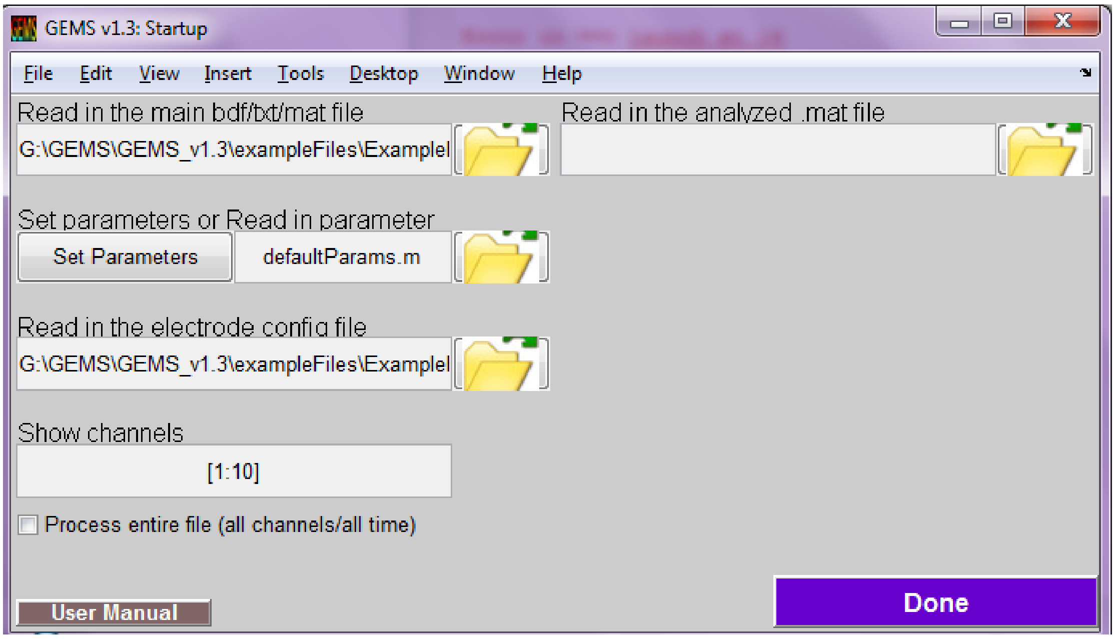

Figure 7: Select files.

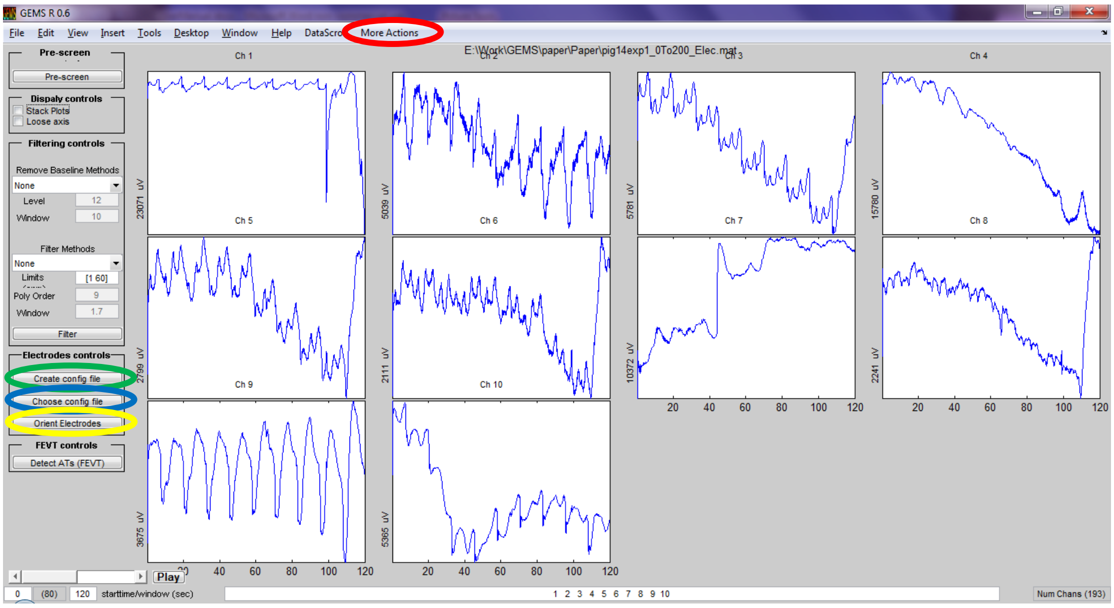

Figure 8: GEMS figure.

7 Electrode configuration file

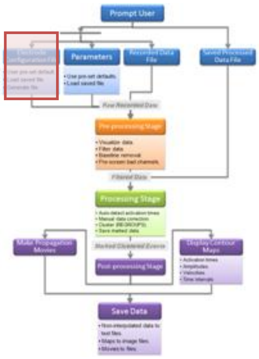

1. You can either read the '.txt' file or create it.
2. If you are using the default example files, then the electrode configuration file is already defaulted in the start-up figure.
3. To read a configuration file press the *Choose config file* button (green circle in Figure 8) and locate the electrode configuration file on your machine.
4. To create a configuration file press the *Create config file* button (blue circle in Figure 8). A window should appear as shown in Figure 9.

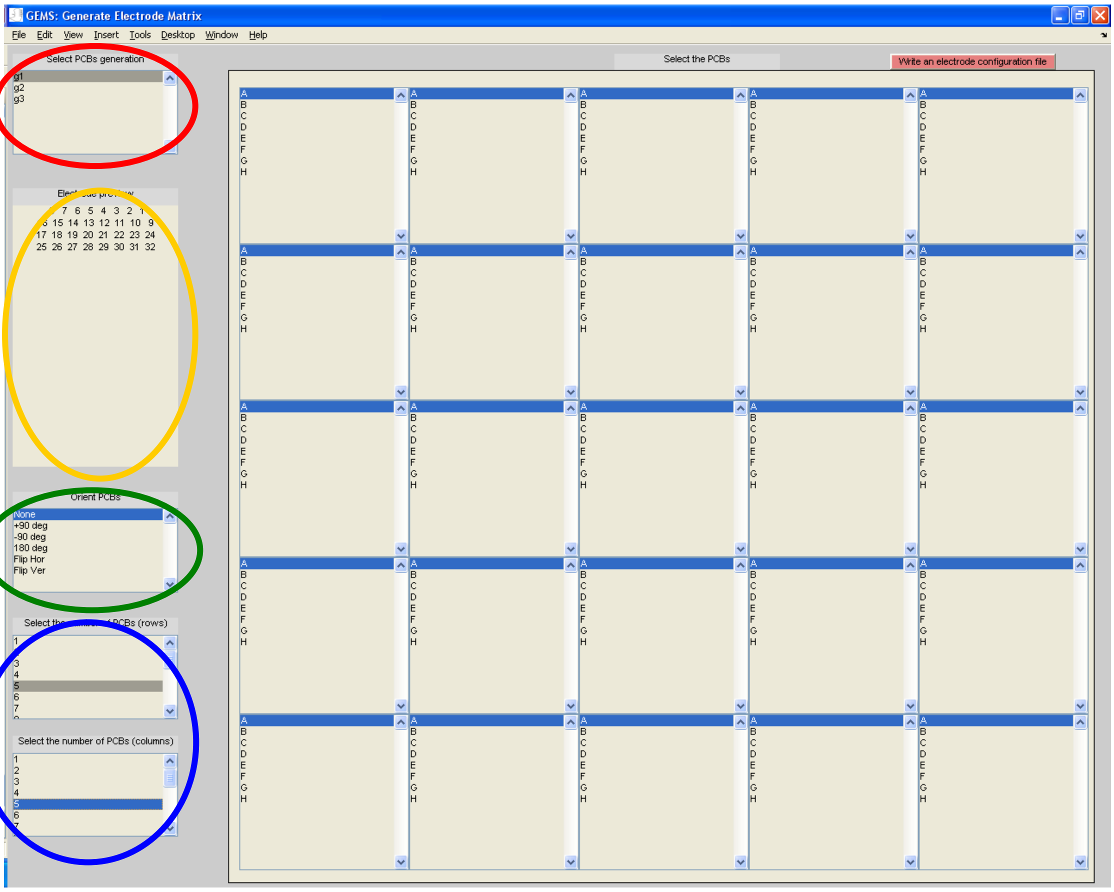

Figure 9: Generate Electrode Configuration File.

5. In the figure, select which PCB generation you want from the *Select PCBs generation* list box (red circle in Figure 9). A preview of a single PCB orientation will appear in the *Electrode preview* window below it (yellow circle in Figure 9).
6. The orientation of the electrodes within the PCBs used can be changed using the *Orient PCBs* list box (green circle in Figure 9). The new orientation will appear in the *Electrode preview* window.
   NOTE: all the other PCBs used for the experiment will follow the same orientation.
7. The user can then select how many PCBs they had in rows and columns (blue circle in Figure 9). The corresponding number of PCBs will appear as list boxes in the figure.

8. Select PCBs from the list boxes based on the orientation used in the experiment.
9. When finished, press the pink button *Write an electrode configuration file*. User will be prompted for a file name, and will be asked if they wish to use this configuration for the main GEMS program. If not then user will be reminded to read an electrode configuration file.
10. To check the orientation of the electrodes press the *Orient Electrodes* button (yellow circle in Figure 8), and Figure 10 will pop-up. You can close the electrode config figure or leave it open.

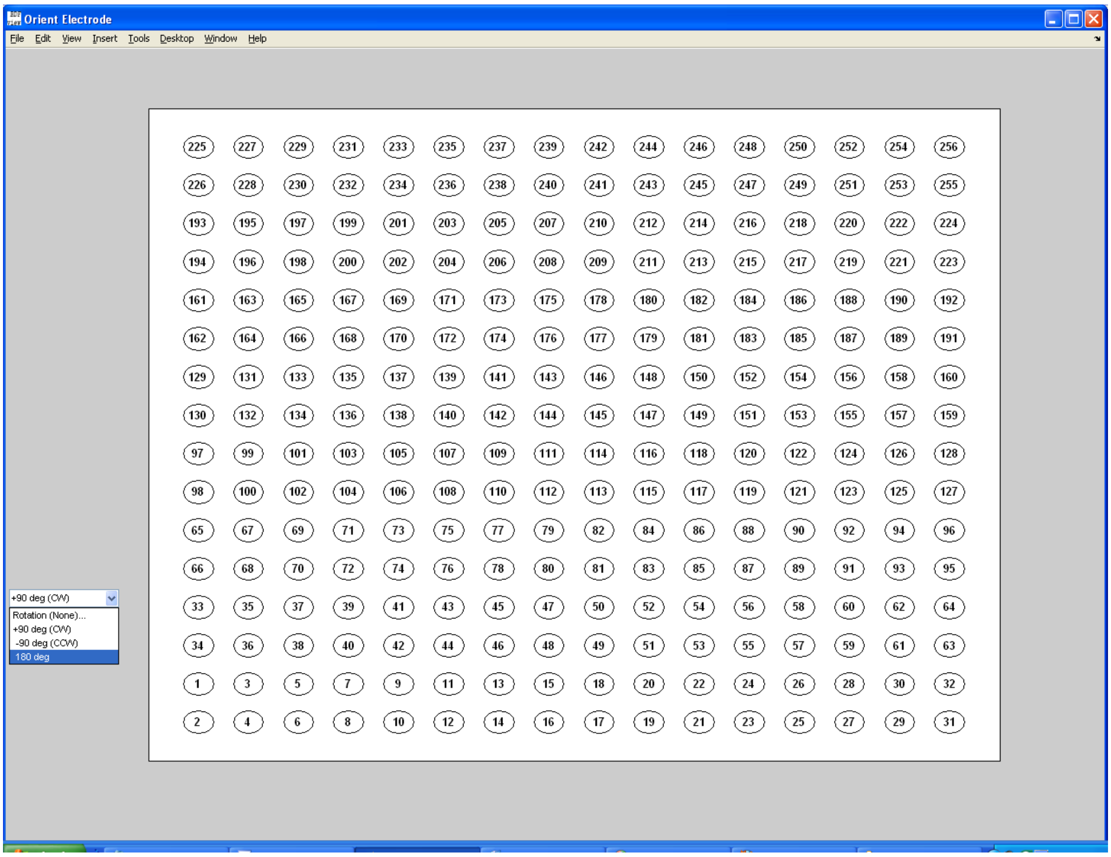

Figure 10: Electrode Configuration.

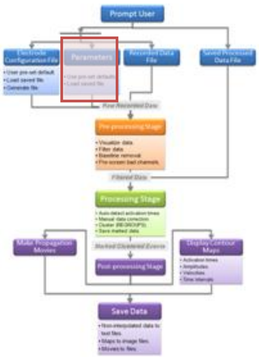

8 Default parameters

1. To set the default parameters: **More Actions – Set Parameters** (red circle in Figure 8). The parameters will be displayed in a figure as shown in Figure 11. Parameters are grouped into different sections and they can be accessed using the buttons on the top of the figure (highlighted in red circle).
2. After you change the parameters, click on the *Change Parameters* button to update the parameters otherwise nothing will be updated. The *Change Parameters* button updates the parameters for the current file you are using in GEMS, if you exit GEMS and re-launch it, it will not go back to these parameters unless you have saved the parameters to a file and read that file back in (next step explains how to save the parameters to a file).
3. To save the list of parameters to a '.mat' file click on *Save Parameters*. Make sure you save the parameters at the end before you exit so it will keep the latest parameters. If you save the parameters to a file and then later on change something in the figure and forget to save it, next time you read the set of parameters they will not be the latest, i.e., pressing the *Change Parameters* is not the same as saving them. If you change some parameters, make sure you press *Change Parameters* before you press *Save Parameters*,
4. To load a set of parameters click on *Load Parameters*.
5. Once done click on *Close Me* button, this will hide the figure.
   NOTE: DO NOT close the figure using the 'X' button at the top right hand corner. If you do by mistake, you will need to re-run GEMS again and risk losing all your current work (mainly marked events).
6. You can also get this figure back from the AT figure: **Other Functions – Set Parameters**.

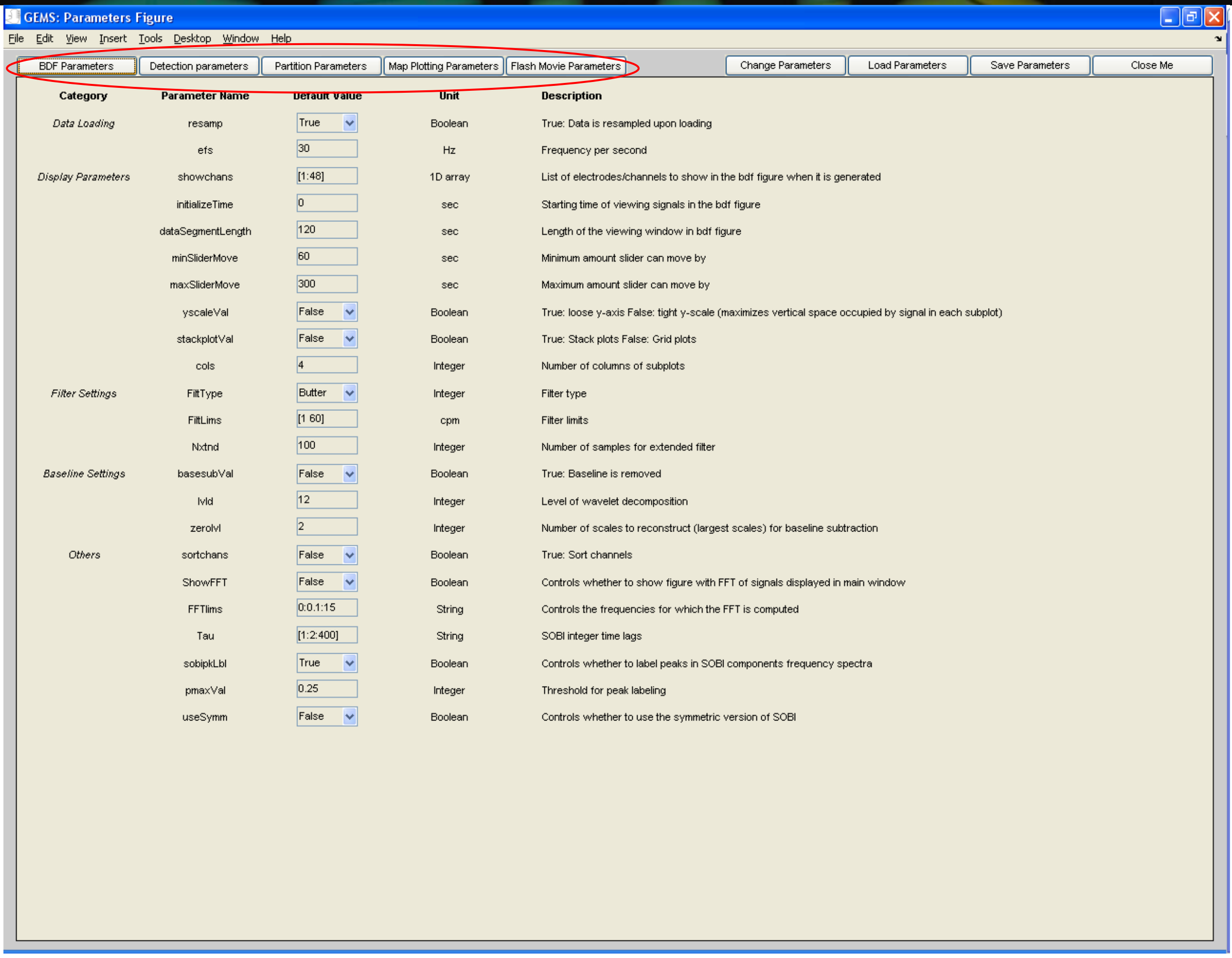

Figure 11: Default Parameters.

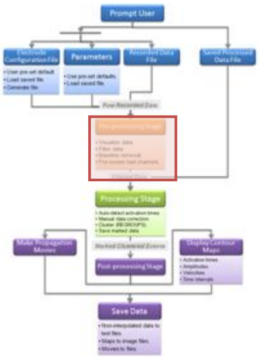

9 Main GEMS figure controls

1. This main figure contains the raw recorded data, as shown with the example in Figure 12.
2. To stack the subplots (plots for each electrode) in one column, select the *Stack Plots* check box (green circle in Figure 12).
3. *Loose axis* checkbox determines scaling of y-axis of plots. When loose-axis is checked all y-axis limits will be set equal to one another. This is useful when comparing the amplitudes across channels.
4. To remove baseline drift, user can select a baseline removal method from the *Remove Baseline Methods* list (red circle in Figure 12). To apply the baseline drift removal and the filtering, user must click on *Filter* to see any changes in the viewed signals.
   NOTE: some baseline removal methods will require the appropriate toolboxes, i.e., DWT requires wavelet toolbox.
5. The drop down menu *Filter* (blue circle in Figure 12) allows the user to select the filter method. The boxes just below it allows you to set the filter limits associated with the filtering methods (note the boxes will lock/unlock depending on the filtering method chosen). For all filter types, except CWT Stopband, *Limits* indicates the filter's passband. For CWT Stopband, *Limits* indicates the stopband frequencies. For the *WT filters, you can enter multiple *Limits*, e.g. [0:20, 40:60] with CWT Passband passes all frequencies in the range of 0-20 and 40-60 cpm (blocking the 20-40 cpm band). You can NOT use this feature when the filter type is butterworth. The current filter types are:

   -butterworth: 2nd order butterworth filter. Filter limits specify b

   -DWT: dwt "denoising' filter (typically not used)

   -CWT Passband: cwt "denoising' filter (mother wavelet is 'morlet'), the limits entered in

   -CWT stopband: cwt "denoising' filter (mother wavelet is 'morlet'), specify stopband. CWT has the advantage of an effectively infinitely sharp passband, but is quite slow. For ~20 channels of 1800 samples, expect to wait a few seconds.

   -SG Filter: smoothing filter where polynomial order and window size can be set

   -Moving median: baseline removal method where the window size is specified.

   NOTE: some filtering methods will require the appropriate toolboxes, i.e., DWT requires wavelet toolbox.
6. To apply the baseline drift removal and the filtering, user must click on *Filter* to see any changes in the viewed signals.
7. Channel box (black circle in Figure 12). Enter the channels in this box that you wish to display. You can enter MATLAB style lists, such as 1:20, 40:48 or 1, 2, …, 20, 40, 41, …, 48 or 60:5:80 (display every 5th channel starting from channel 60 and ending at channel 80). The number in parentheses indicates the total number of channels available for viewing. Note that the channel list will be automatically sorted, excluding any duplicated or invalid

channels, if the sortchans parameter is set to 1. If sortchans is set to 0, channels are displayed in the order specified.

8. The *Play/Stop* button controls automation of scrolling through or playing back data.
9. To change the time for the display window: change start time and the length of the displayed window.
   NOTE: you can use the slider to move through time (start time in purple circle, time for the subplot/window is in grey circle in Figure 12).
10. The **Data Scroll** menu (orange circle in Figure 12):
    a. **Fwd:** advances time by 60 sec (or to max allowable)
    b. **FastFwd:** advances time by 300 sec (or to max allowable)
    c. **Rewind:** decrements time 60 sec (or to min allowable = 0 sec)
    d. **FastRewind:** decrements time 300 sec (or to min allowable = 0 sec)
    e. **Playback params:** controls the parameters when “Play” is set to on.
       - Increment is the amount by which the time is advanced before displaying next chunk of data (in sec). Default is 60 sec
       - Pausetime is the amount of time to wait before trying to display the next data chunk (in sec). Default is 2 sec.
11. The **More Actions** menu (light blue circle in Figure 12):
    The relevant options are:
    a. **Set Parameters:** bring up the parameter figure.
    b. **Read analyzed data:** read a ‘.mat’ file which contains analysed data.
    c. **User Manual:** a call to this user manual.

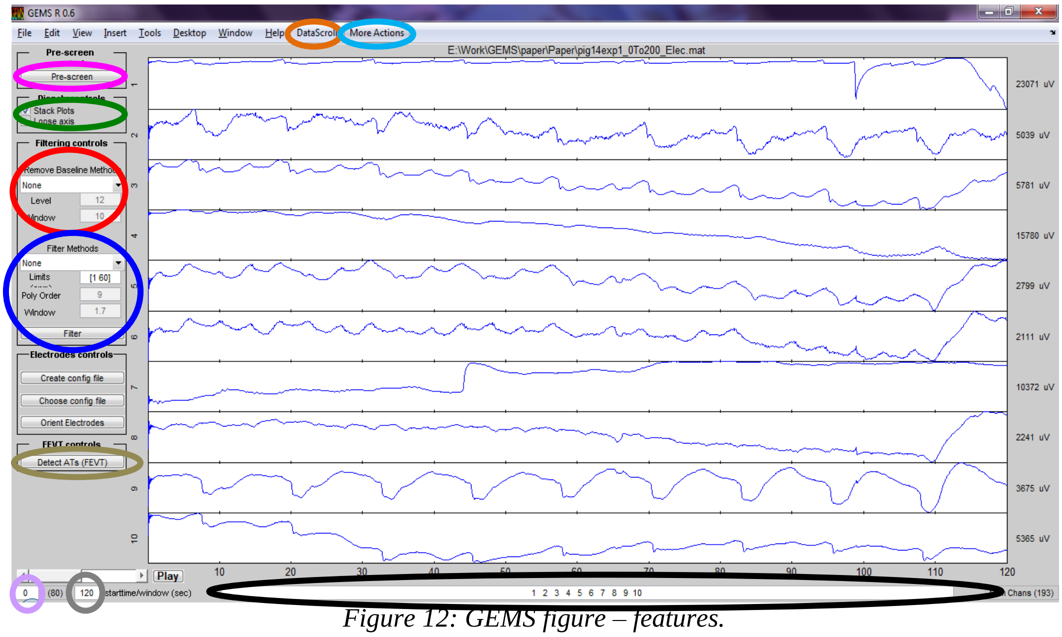

Figure 12: GEMS figure – features.

10 Pre-screen

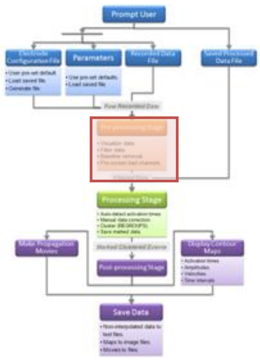

1. To eliminate bad channels from being considered for marking, click on the *Pre-screen* button on the GEMS figure (pink circle in Figure 12).
2. A figure will pop up. On the left hand side, you can select multiple channels to view at one time (red circle in Figure 13). You can achieve that by clicking on a channel number, e.g., channel 50, hold down the shift key and select another channel number, e.g., channel 80, you should see all the channels between 50 and 80 are selected. If you want to select scattered channels, then hold down the ctrl key while clicking on channel numbers.
3. The channels will be displayed in the GEMS figure. If you are happy with the channel (i.e. good channel) then click on the channel number check box on the right hand side of the pre-screen figure (green circle in Figure 13).
   NOTE: if the channel is somewhat good, or if you are unsure about it, then select it, you can always delete the markers later, BUT you CANNOT add channels later.
4. If you want to select all channels, then unselect the bad ones, then press the *Select all channels* button (black circle in Figure 13).
5. If you want to unselect all channels, press the *Unselect all channels* button (purple circle in Figure 13).
6. At the bottom of the figure (pink circle in Figure 13) there is an edit box which has the list of the original channel list in the GEMS main figure just before the pre-screen button was pressed. You can also edit this box by either adding or removing channel numbers, it is not connected to anything, it is just made for manual editing, like a scrap box for the user to type channels in may be if they are unsure of it or want to revisit it later.
7. Once you are done click finish (blue circle in Figure 13) and WAIT for the GEMS figure to update itself to include all the good channels. The total number of selected channels will appear on the top right hand side of the figure (yellow circle in Figure 13).
8. Make sure that the time selected in the GEMS figure is what you want to include for the detection, i.e. the time for the window should be the duration of the experiment if you want to process the entire file.

NOTE: You can completely bypass this pre-screening step, by just typing the number of channels you are interested in processing in the GEMS main figure (black circle in Figure 12), for example: 1:256 will plot channels 1 to 256.

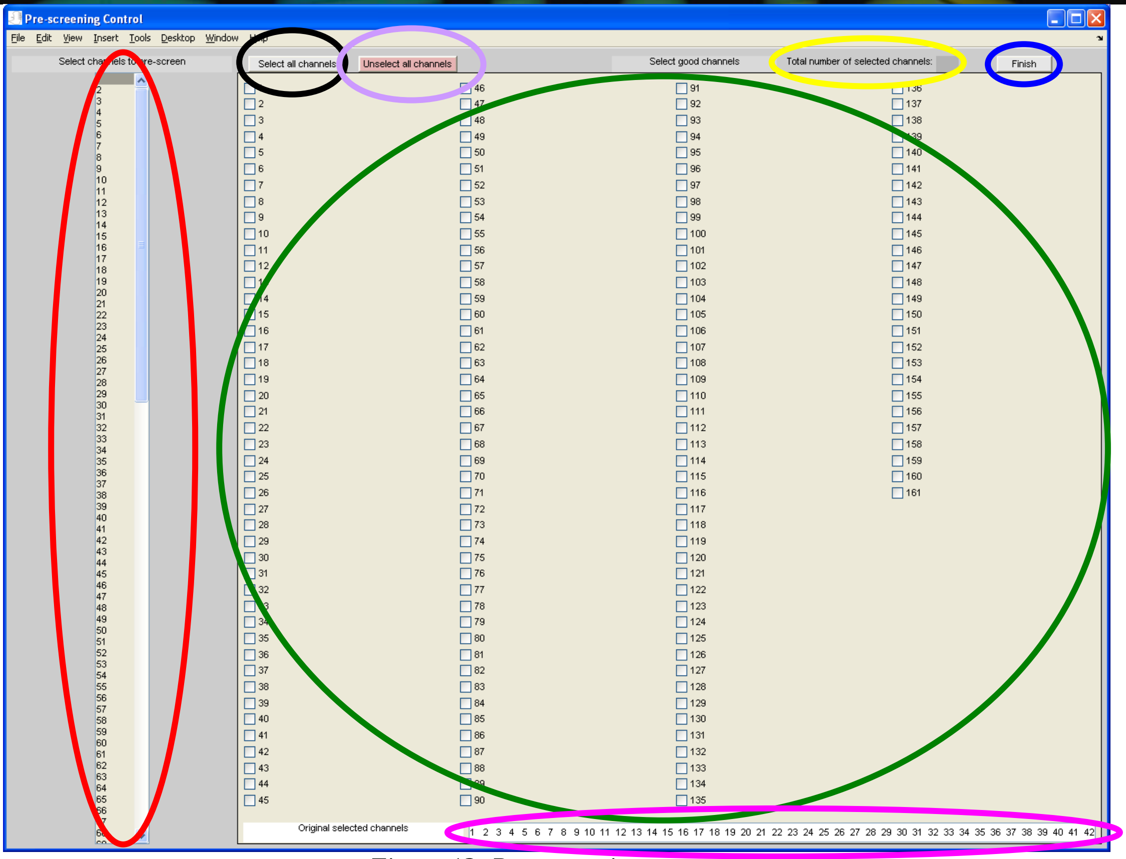

Figure 13: Pre-screening.

11 Auto-marking

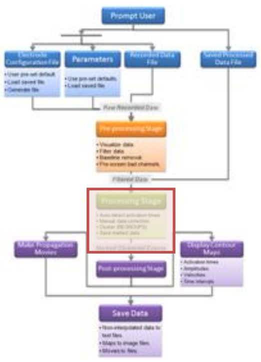

1. To auto detect activation times (ATs), in the main GEMS figure press the *Detect ATs (FEVT)* button on the main GEMS figure (light brown circle in Figure 12). This will detect ATs for the viewed channels and viewed time.
2. Wait until the algorithm finishes detecting events. When it's done, the AT figure will pop up (see Figure 14(a)).
   NOTE: don't alter anything in the main GEMS figure after you do this. It might alter other important things and cause problems.
3. If you check the *Plot clustered waves* (orange circle in Figure 14(a)) the plots will change displaying the clustered waves in different colours (Figure 14(c)). If you check the *Plot wave numbers* then the wave numbers will be displayed on top of each marked event. Unclustered ATs (or orphans) will be plotted in green squares with red outlines.
4. To view all the marked events press the *View ALL markers* button,
   NOTE: for large files this might take a long time (red circle in Figure 14(b)).
5. To remove all the plots click on the *Remove current plots* button (black circle in Figure 14(b)). In the command window it will indicate which axes it's up to in the deleting stage.
6. If you want to remove the markers of an entire channel, then type in the channel or channel numbers in the *Type channel numbers to delete their markers* edit box (purple circle in Figure 14(a)) and then press the yellow *Delete Markers* button.
7. If you want to remove the markers of channel(s) based on amplitude lower and/or upper bounds, then type in the channel or channel numbers in the *Type channel numbers to delete their markers using amplitude limits* edit box (light green circle in Figure 14(a)). Then enter lower and/or upper bounds in the appropriate boxes and then press the *Remove Markers* button.
8. *Plot(x,y,t)* button generates an x,y,t plot of the autodetected ATs as shown in Figure 15. Essentially it plots activation times on the z-axis above the physical location of the corresponding channel. The different coloured points correspond to individual slow wave events. That is, the software incorporates an algorithm to discern which points belong to each individual slow wave.
9. To export the visible electrograms select *Export* button (yellow circle in Figure 14 (a)). This will generate a figure which will contain the axes you are looking at in the AT figure and save it to a '.tif' file. User can set the size of the figure to export by setting the % value (green box). If you want to generate electrogram figure with no markers, select the *Don't Plot Markers* checkbox.
10. To unify the scale of all the y-axes displayed in the AT figure check the *Global y-axes* check box on the AT marked figure (Figure 14 (a)).
11. **Other Functions** menu bar**:**
    a. **Export Unclustered Data to .txt File:** exports the activation times, amplitude and width of the downward stroke to '.txt' file of the unclustered marked activation times.
    b. **Export Markers to .txt File (SmoothMap):** exports the markers to '.txt' file which can be read into SmoothMap.

c. **Export Markers to .mat File:** exports the markers to '.mat' file which can be read into GEMS. This is the same as pressing the *Save Markers* button.
d. **Cluster Waves (REGROUPS):** forces GEMS to recluster the current marked ATs.
e. **Orient Electrodes:** changes the orientation of the electrodes. This will open another GUI figure window which allows you to orient the electrodes as desired. This effects the display only of latter figures to be generated. For example, if you want to see on your computer screen the gastric slow wave propagating as if you were looking down at the test subject, head at top, you can choose the appropriate options (rotate/flip horz/flip vert) to accomplish this. This also typically requires knowledge of how the electrode was oriented on the test subject, so keep careful notes.
f. **Auto-detect bad channels:** under development.
g. **Re-detect ATs:** this will launch execution of FEVT algorithm. The figure will be re-plotted with the corresponding ATs for each channel.
h. **Re-filter:** this will refilter the signals. Change the filtering parameters as desired in the parameter figure before selecting this option.
i. **Statistical Analysis:** refer to Section 15.
j. **Flashlight Display:** creates '.avi' movie files. This is the same as pressing the *Flashlight Movie* button.
k. **Set Parameters:** launches the parameters figure.
l. **User Manual:** launches the user manual.

NOTE: If you change any settings/parameters in the main GEMS window, you need to re-launch the AT marked figure from the main window to reflect these changes. If you only change the detection parameters using the Parameters figure then you do not need to re-launch the AT marked figure.

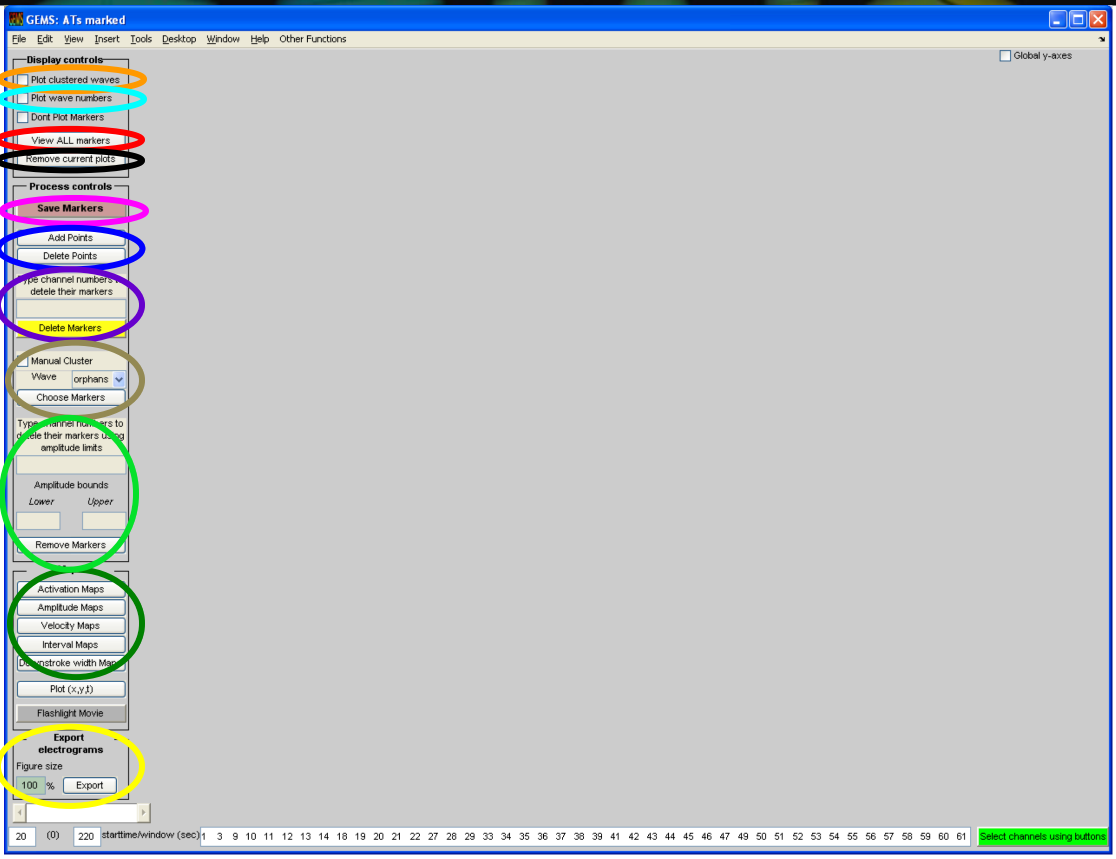
GEMS: ATs marked
File Edit View Insert Tools Desktop Window Help Other Functions
Global y-axes
Display controls
Plot clustered waves
Plot wave numbers
Dont Plot Markers
View ALL markers
Remove current plots
Process controls
Save Markers
Add Points
Delete Points
Delete Markers
Manual Cluster
Wave
orphans
Choose Markers
Amplitude bounds
Lower
Upper
Remove Markers
Activation Maps
Amplitude Maps
Velocity Maps
Interval Maps
Plot (x,y,t)
Flashlight Movie
Export electrograms
Figure size
100
%
Export
20
(0)
220
starttime/window (sec)
Select channels using buttons

(a)

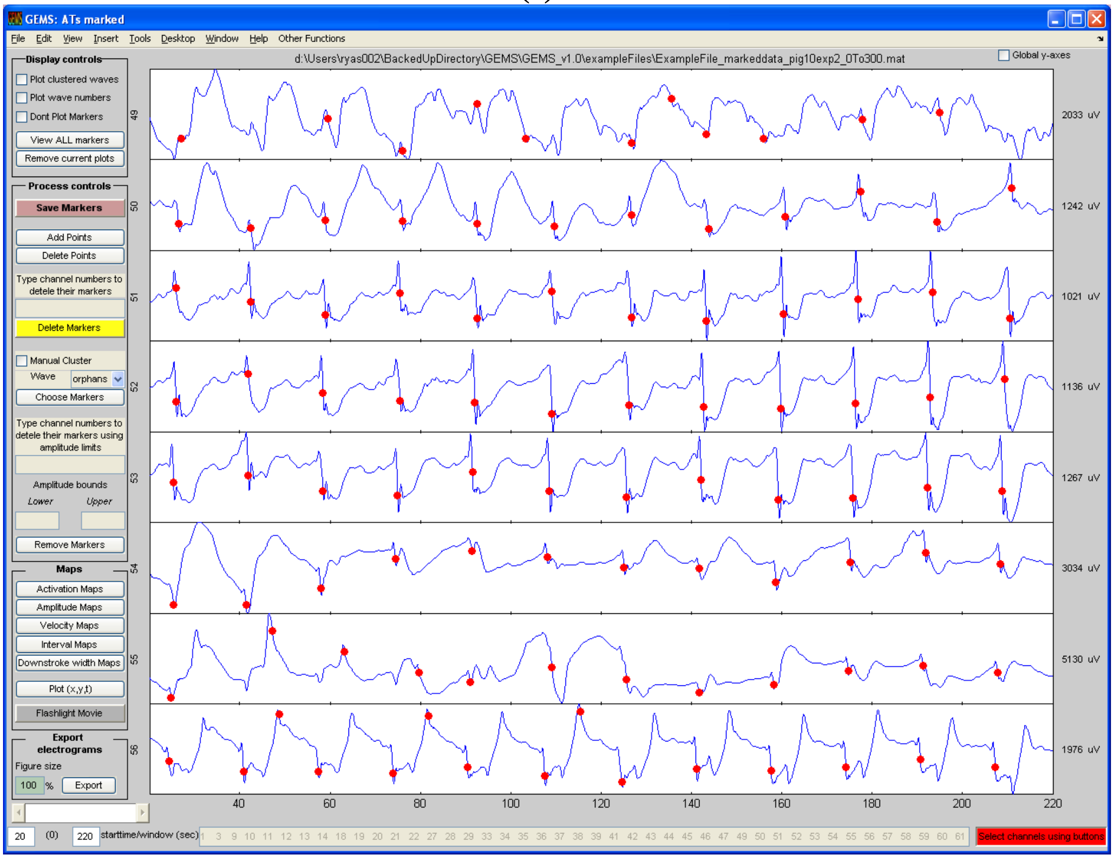
GEMS: ATs marked
File Edit View Insert Tools Desktop Window Help Other Functions
d:\Users\ryas002\BackedUpDirectory\GEMS\GEMS_v1.0\exampleFiles\ExampleFile_markeddata_pig10exp2_0To300.mat
Global y-axes
Display controls
Plot clustered waves
Plot wave numbers
Dont Plot Markers
View ALL markers
Remove current plots
Process controls
Save Markers
Add Points
Delete Points
Type channel numbers to detele their markers
Delete Markers
Manual Cluster
Wave
orphans
Choose Markers
Type channel numbers to detele their markers using amplitude limits
Amplitude bounds
Lower
Upper
Remove Markers
Maps
Activation Maps
Amplitude Maps
Velocity Maps
Interval Maps
Downstroke width Maps
Plot (x,y,t)
Flashlight Movie
Export electrograms
Figure size
100
%
Export
49
50
51
52
53
54
55
56
2033 uV
1242 uV
1021 uV
1136 uV
1267 uV
3034 uV
5130 uV
1976 uV
40
60
80
100
120
140
160
180
200
220
20
(0)
220
starttime/window (sec)
Select channels using buttons

(b)

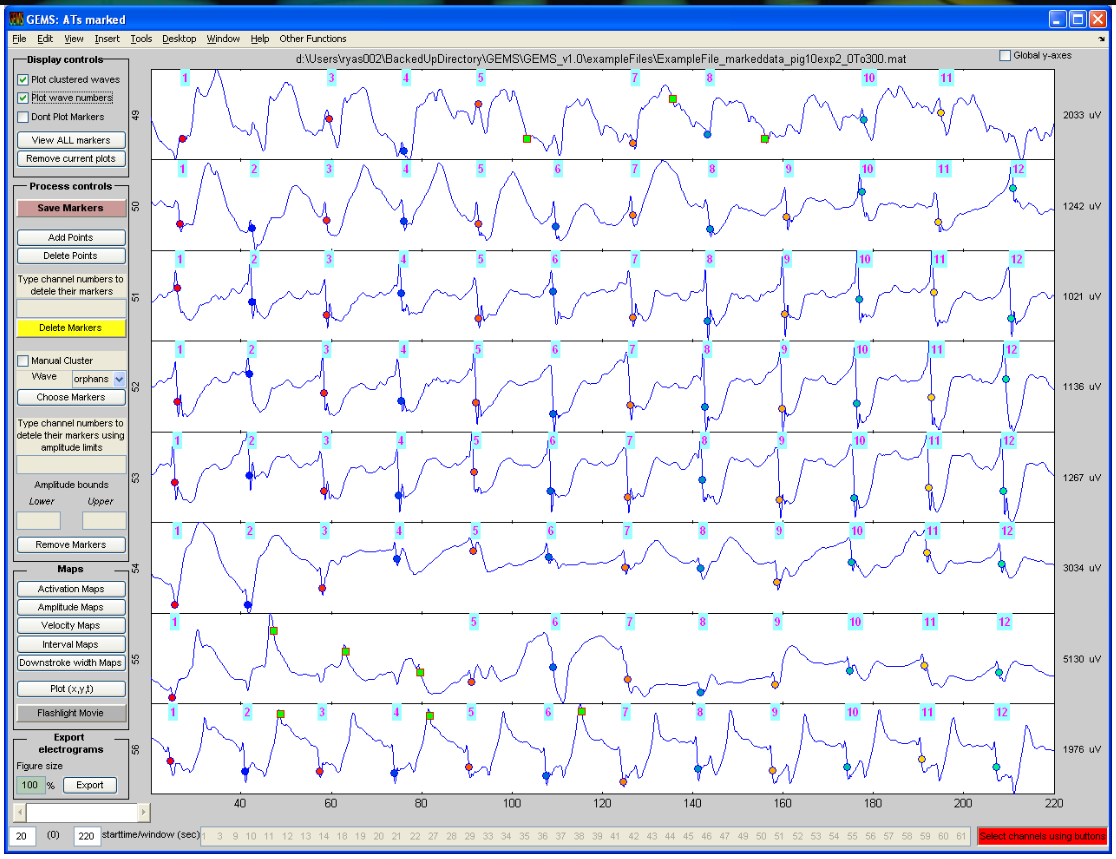

(c)

Figure 14: Auto-markers.

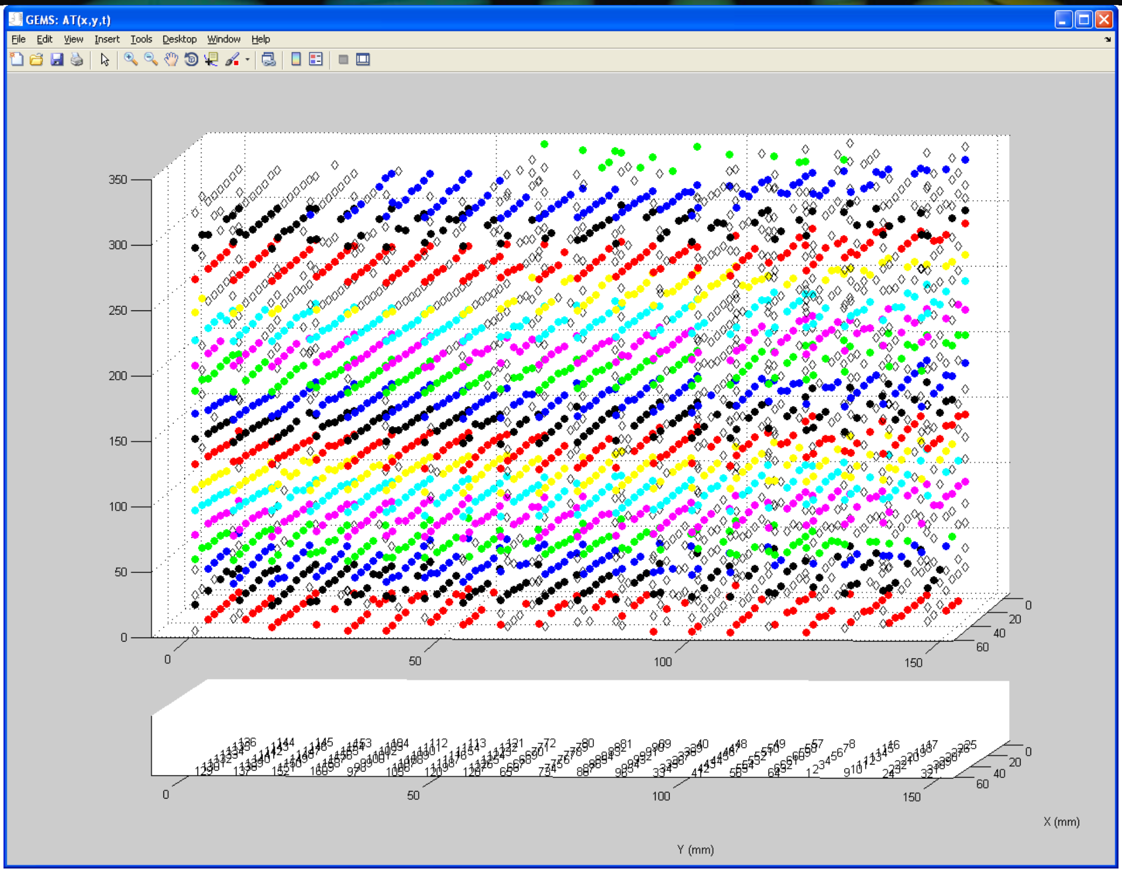

Figure 15: Plot x,y,t.

12 Post-processing

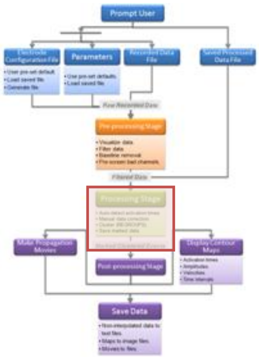

1. To check the markers closely, click on the green button (*Select channels using buttons*) at the bottom right of Figure 14 (a). OR you can manually type the channel numbers in the channel listbox at the bottom of the AT marked figure.
2. A figure will pop up with buttons. The *Select channels using buttons* will turn red to indicate that you are post-processing.
3. The green buttons on the Channels Map figure (Figure 16) refer to the channels that were auto-marked. If user has discarded any channels in the pre-screening stage, the buttons of these discarded channels will be shown here in grey and they will be locked.

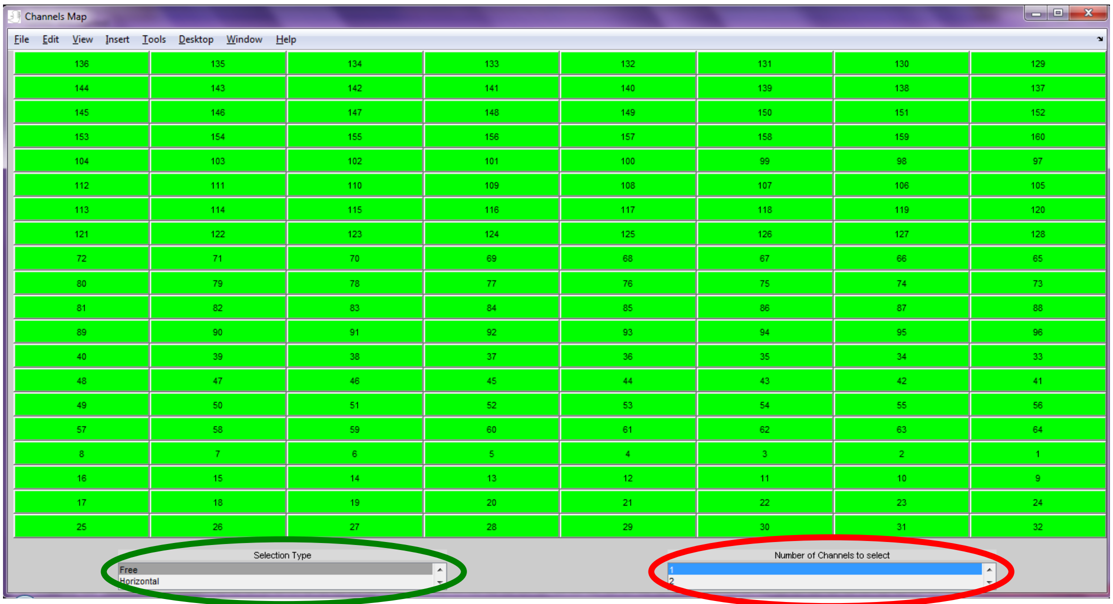

Figure 16: Channels map - post-processing.

4. You can select the channels either freely, horizontally or vertically in the *Selection Type* list box (green circle in Figure 16).
5. If you select vertical or horizontal you can specify how many channels to select from the *Number of Channels to select* list box (red circle in Figure 16).
6. As you select the channels to view, the plot will change on the ATs marked figure. You can then delete false markers or add more markers using the *Delete Points* and *Add Points* buttons (dark blue circle in Figure 14).
7. When you click the *Add Points* button the button will change to *Adding Points* in red (Figure 17(a)). To add markers, point where you want to add the marker with the mouse and use the left mouse click. Use the right mouse click to mark the last point you want to add and that will automatically finish the adding process. If you are in the clustered view, the new point will be added to orphans.
   NOTE:
   - As you click on *Add Points* button the *Delete Points* will be disabled.
8. When you click the *Delete Points* button the button will change to *Deleting Points* in pink (Figure 17(b)). To delete markers, point to the marker you want to delete with the mouse and use the left mouse click. Use the right mouse

click to delete the last marker and that will automatically finish the deleting process.

NOTE:

- As you click on *Delete Points* button the *Add Points* will be disabled.

9. To manually cluster events, check the *Manual Cluster* checkbox (brown circle in Figure 14(a)). Select which wave you want to cluster from the drop down box, once you select that, click on *Choose Markers*. This will lock the wave drop down box to ensure you finish one wave at a time. You can then start selecting which points you want to add to that wave. Select using the left click, last point to select use the right mouse click, this will select the last point and also stop the selection process. As you are selecting, the points will turn into a pink box to show you the points you selected as shown in Figure 18. The code will ensure you have a single point per wave number per channel, it will automatically set the older marker to an orphan if double ups occur.
10. If you are clustering points and want to add them to a new wave number, ensure that the new wave number is a successive number from the current maximum wave numbers, i.e., if currently you have 5 waves, then the next wave number to manually cluster should be 6, do not skip numbers, i.e., you can't start selecting wave number 10.

NOTES: the manual cluster option can also be changed in the main default parameter figure, if you uncheck the *Manual Cluster* box, GEMS will autocluster, so if you don't want GEMS to autocluster keep this box checked. REMEMBER, if you do that save the parameter file so next time when you read it back in GEMS knows not to auto-cluster.

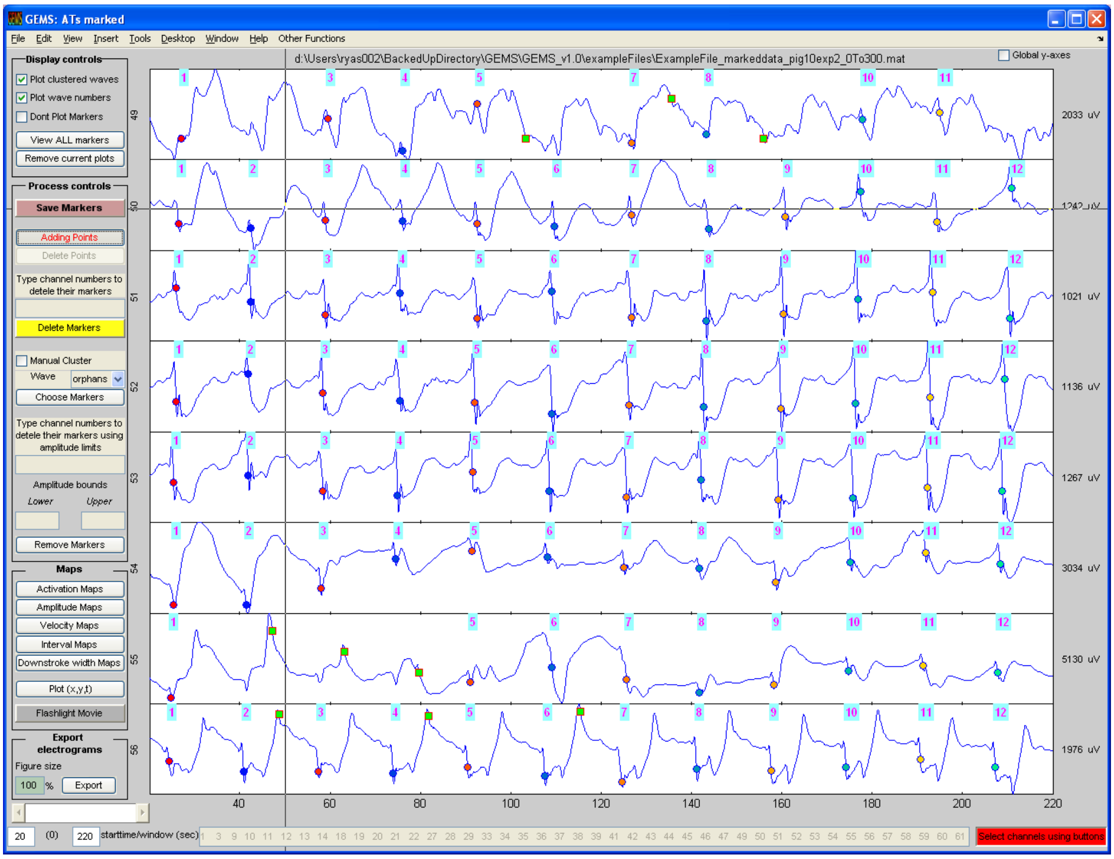

(a)

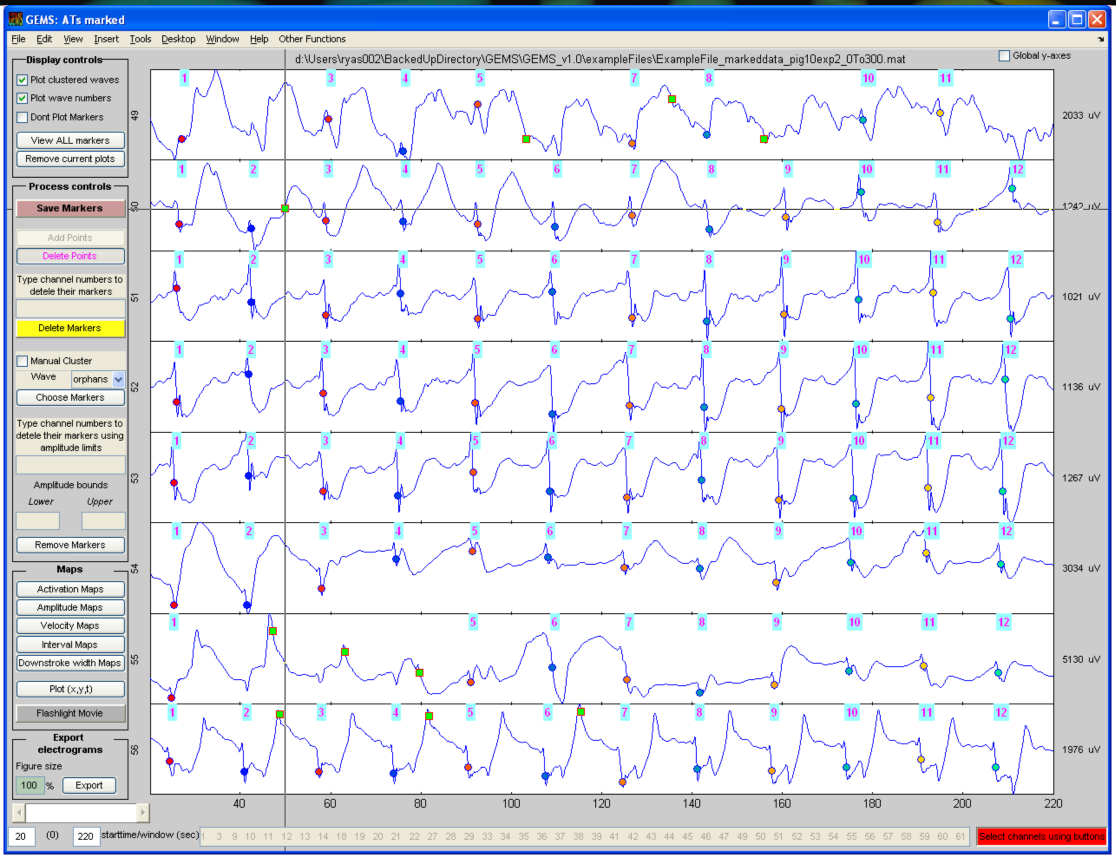

(b)

Figure 17: Adding and deleting markers.

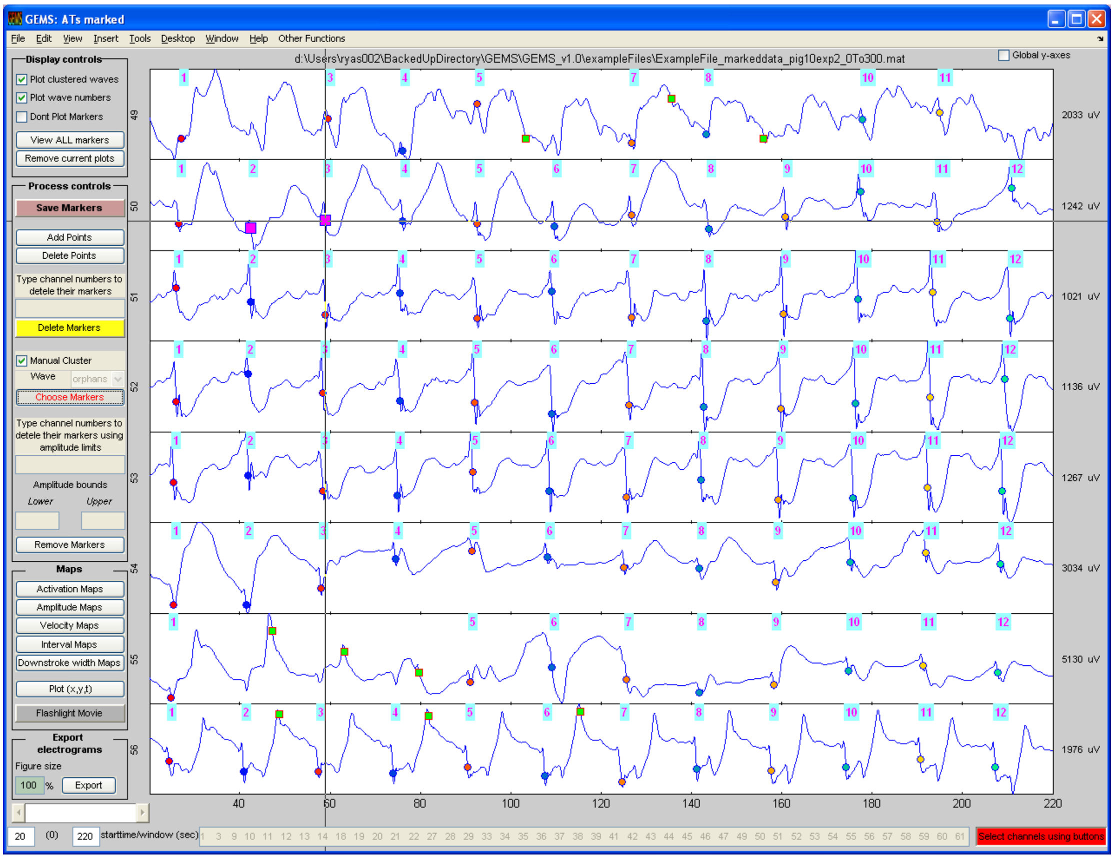

Figure 18: Manual Clustering.

13 Time/Amplitude/Velocity/Interval/Downstroke width maps

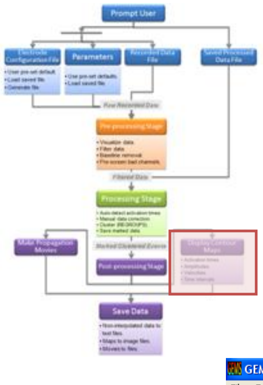

1. You can view the activation times, amplitude, interval and velocity maps by using the buttons on the ATs marked figure (green circle in Figure 14): *Activation Maps*, *Amplitude Maps*, *Interval Maps*, *Velocity Maps* and *Downstroke width Maps*.
2. Corresponding figures with each of the buttons will pop up as shown by the activation times example in Figure 19.
3. Black points/circles represent electrodes for which a data point exists for that particular wave. Red circles represent electrodes which did not record an activation time for that wave.

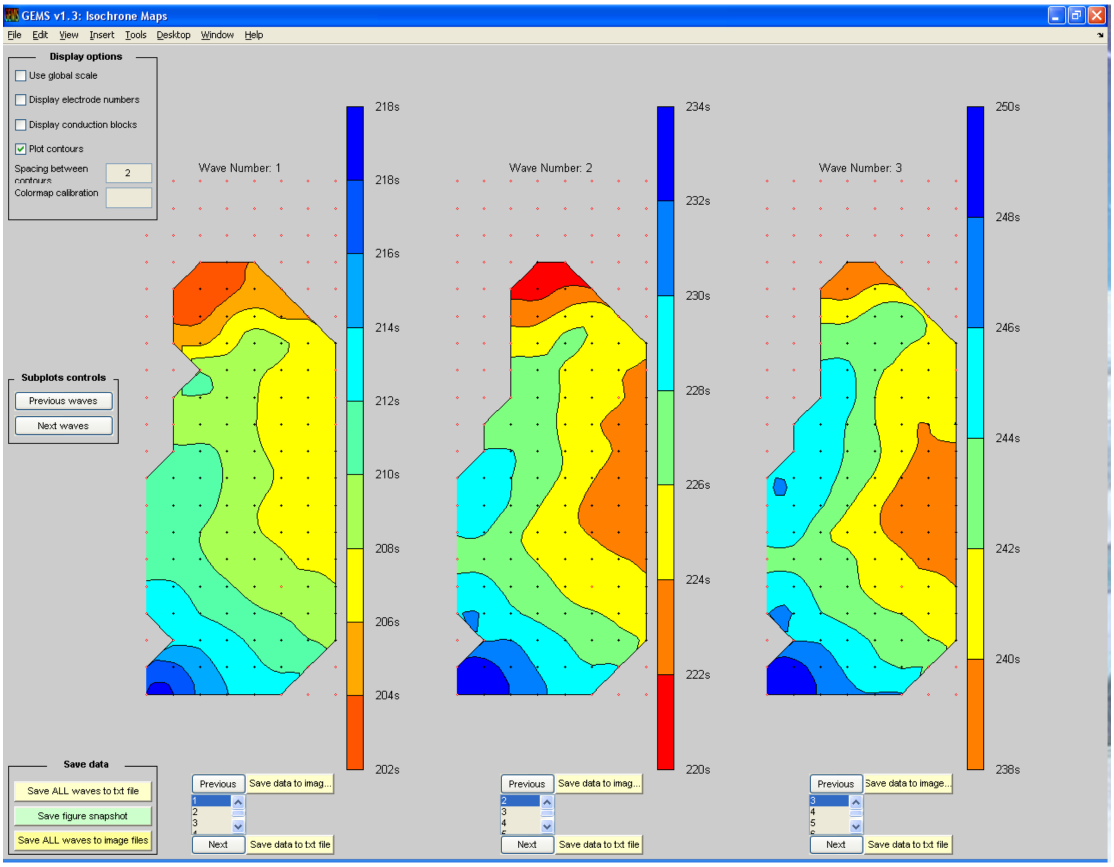

Figure 19: Activation maps.

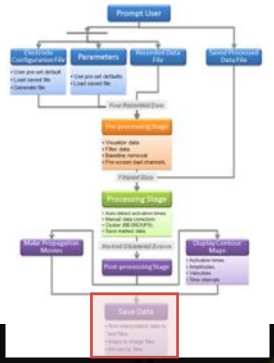

4. Use the *next/previous* buttons under each plot to move through the different wave numbers/events. You can use the *next waves* and *previous waves* buttons to increment/decrement all figures. Also you can use the wave number list box to manually select which wave number you want to look at in each plot.
5. You can globalise the scale of all the three figures by checking the *Use global scale* checkbox (all figures will have a unified scale).
6. You can display the electrodes onto the maps by clicking on the *Display electrode numbers* checkbox.

7. You can display the conduction blocks by selecting the *Display conduction blocks* checkbox. Conduction blocks will be displayed as grey patches.
8. You can switch between contours and patch plots using the *Plot contours* checkbox. If the checkbox is selected, for the AT maps and interval maps you can set the spacing between the contours and the calibration colormap value (For time if you want to force the contour plot colormap to colorcode according to this amount of time, otherwise, leave blank to let matlab draw the contours as it pleases). For the other maps you can set the number of contour lines.
9. You can save the data (uninterpolated) into text files by pressing the *Save data to txt file* buttons under each plot OR you can save all the data (for all the waves) into a txt file by pressing the *Save ALL waves to txt file* button (Figure 19).
10. You can also save the displayed figures to image files using the green *Save figure snapshot* button (Figure 19).
11. OR you can save all the waves to separate image files (1 image file for each wave) by pressing the yellow *Save ALL waves to image files* button, or you can save the current wave per subplot to an image file using the *Save data to image* button under each subplot.

    NOTE: log text files are saved with every image file containing the list of parameters used to generate the figure, this will allow the users to re-generate the figures at a later time with ease.

14 Flashlight movie

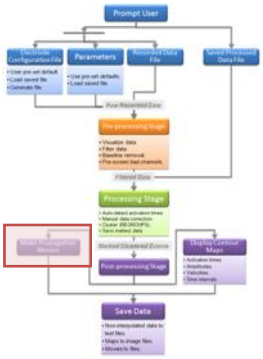

1. From the parameters figure you can choose if you want the movie to be black and white or colour, the starting and ending time for the movie, whether you want to manually group the different wave numbers and associate them with colours.
2. You can either create an ‘avi’ file, or save the frames to ‘tif’ images then combine them to make a movie of desired extension using the *flashlightMovieFileType* parameter. Saving to the ‘tif’ files option can be used when you have a long data recording and MATLAB runs out of memory when creating the ‘avi’ file. Creating the ‘tif’ files might take some time.
3. To create a flashlight movie, press the *Flashlight Movie* button (yellow circle in Figure 14(a)). A new figure will pop-up (Figure 20(a)). The figure represents the electrode configuration map with the marked events moving through time. The current or counter time (depends on user choice in the parameters figure) will increment at the top of the figure as the movie plays. Once it is done, a new figure will pop-up which will allow the user to re-play the movie, fast forward etc.
4. If you selected manual colour assignment for the waves to generate the movie, a figure will pop-up (Figure 20(b)). To manually group the wave numbers and associate them with colours, type the number of colours you want in the *Number of Colours* box then hit enter. The number of colours will be generated automatically on the right and next to each colour an edit box will appear. Enter the wave numbers you want to group into each colour as shown with the example in Figure 20(b), when you are done press *Done*. A figure will appear and show the movie being created.
5. To create movies with half of the screen containing information about the movie set the flashlightWithInfo parameter to true. If you use the black and white option with this setting, the movie will come with red as default.
6. If you want to mark the electrodes which contain no detected ATs in the time period of the movie, set flashlightMarkChanls parameter to 2 in the Parameters Figure. If you want to mark the electrodes which contain no detected ATs at all then set flashlightMarkChanls parameter to 3.

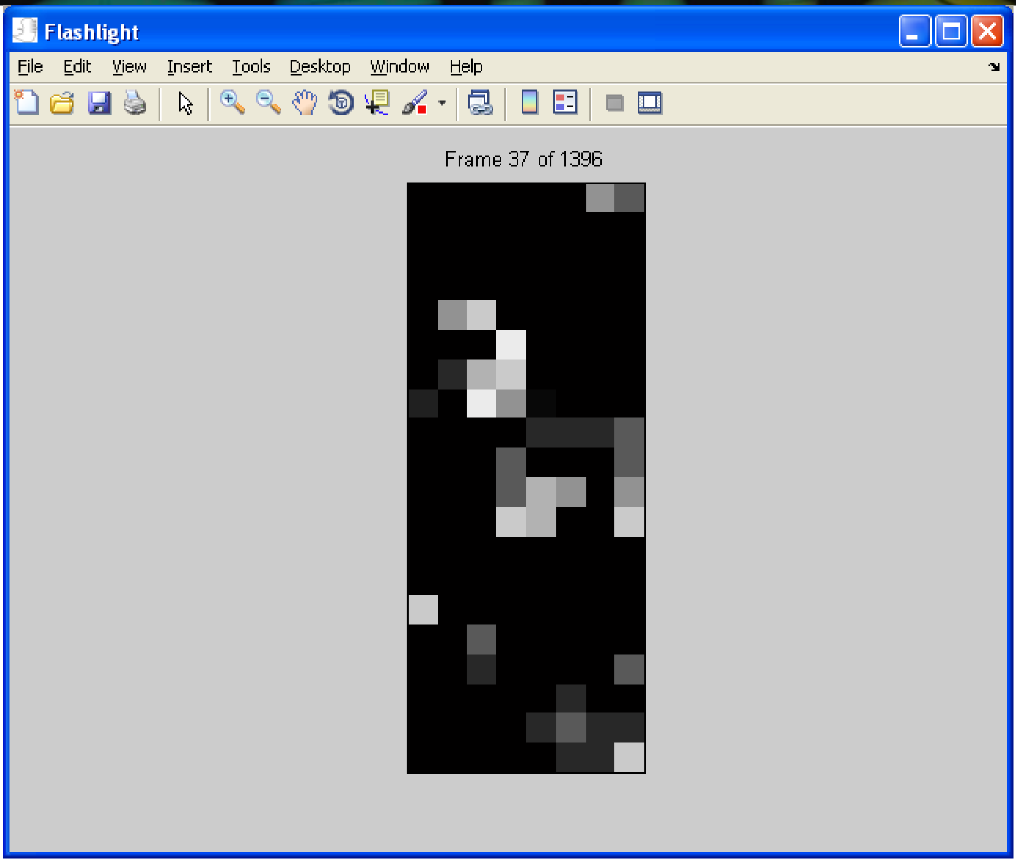

(a)

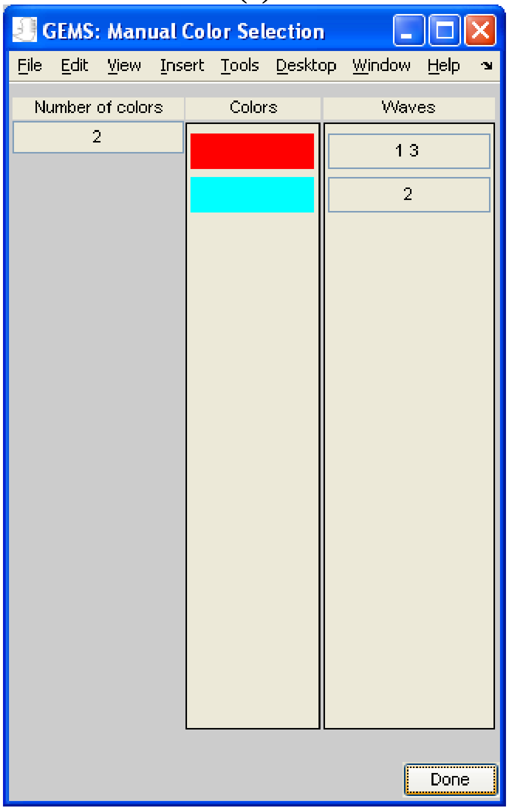

(b)

Figure 20: Flashlight movie.

15 Statistical analysis

1. To calculate the mean and STD for group of electrodes, select **Other Functions - Statistical Analysis** from the ATs marked figure. A new figure will pop up as shown in Figure 21.
2. In Figure 21, the electrode configuration is shown in a form of buttons. To group electrodes, user can click on the desired buttons and then enter a group name in the Group Name edit box then press the *Group* button (green circle in Figure 21). If user presses the *Group* button without entering a group name, an automated group name will be generated. The created groups will be displayed in the *Group List* listbox.
3. To calculate the average and std (AT, amplitude and velocity) for the created groups, user can select single or multiple groups (by holding the shift of ctrl button while selecting) from the *Group List* listbox, and the desired wave numbers from the *Waves* listbox (blue circle Figure 21).
4. To calculate the average and std (interval) for the created groups, user can select single or multiple groups (by holding the shift of ctrl button while selecting) from the *Group List* listbox, and the desired wave numbers from the *Waves* listbox in the Interval section.
5. To save the created groups to '.mat' file so it can be re-read, press on the *Save Groups* button.
6. To load a saved group, press the *Load Groups* button. User will be prompted for a '.mat' file
7. To save the displayed results into a text file, press the *Save Stats to txt* button.

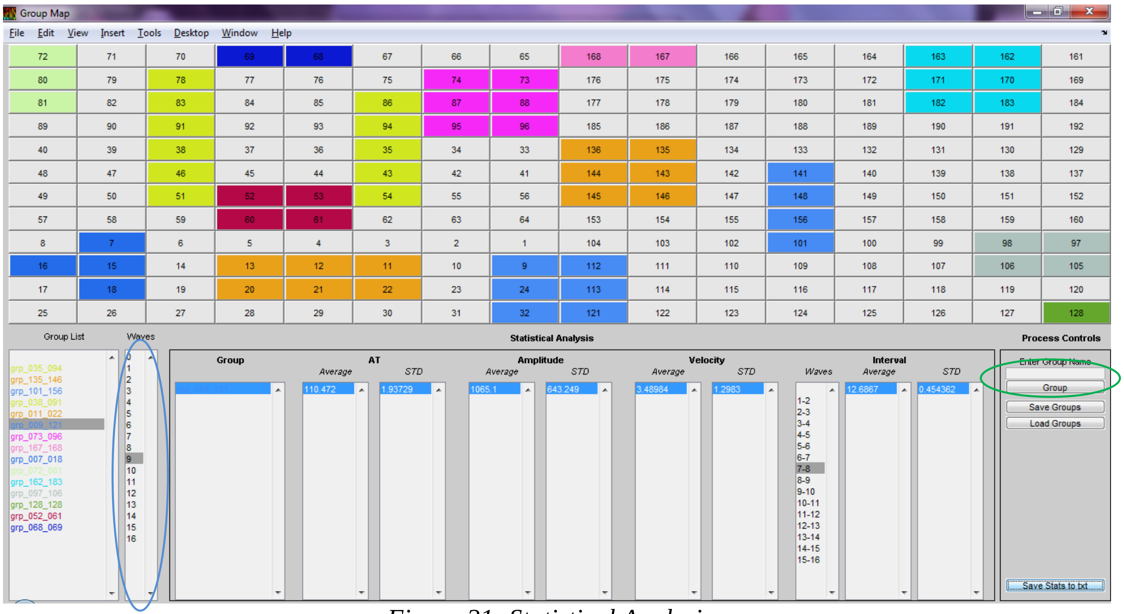

Figure 21: Statistical Analysis.

16 Saving data

1. Data can be saved to a '.mat' file and a '.txt' file: **Other Functions – Export Marks to .mat file**, or/and **Export Marks to .txt file** OR by pressing the *Save Markers* button (pink circle in Figure 14) which will save the data to '.mat' files.
2. The text file is in the format accepted in SmoothMap, the '.mat' file is saved in the format that can then be read again into GEMS for future re-analysis.

17 To re-load analysed data

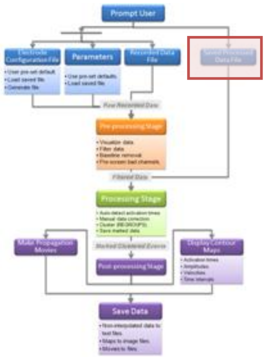

1. Launch MATLAB and GEMS as described above.
2. Read the analysed '.mat' file in the box on the right hand side of the start-up figure and press *Done*.
3. You can plot activation times, amplitude maps, etc, delete or add points as before.

18 Useful Hints

1. In the main GEMS figure, if you are not planning on using the pre-screen and planning on processing all or most channels try this order of operating to save time (it takes time to plot, so every time you change something MATLAB tries to delete all the plots and then plot them again!! So if you have a lot of channels, try to set everything for a single channel then type all channels later, that way you save time):
    a. Type a single channel number (any it doesn't matter) in the channel box at the bottom
    b. Select your baseline removal, filter, time period and if you want to stack or keep the grid view.
    c. Once you are happy with this, then type in all the channel number in the channel edit box at the bottom. That way it only has to delete a single plot then plots the rest after.
2. Due to the way GEMS keeps track of all the associated windows and plotting routines, you cannot open multiple '.bdf/.mat' files. From time to time, if you are clicking around, you may mess up the plotting and callback routines. When this happens, the best course of action is to close GEMS and start fresh by re-launching. Therefore, as soon as you mark events or cluster them, SAVE your work.

19 Glossary of parameters

The table below lists the parameters the user can modify and a brief description of each parameter. These parameters can be accessed from the main GEMS figure and the AT figure as described before. The default values of these parameters are stored in '\usrGUIDefaults\ defaultParams.m'. If you change them for your experiments, make sure you save them into a separate file using the *Save Parameters* button for safe keeping.

Table 1: List of parameters.

Group	Parameter name	Description
BDF Parameters	*resamp*	Set data to be resampled or not upon loading
	efs	Resampling frequency (per second)
	showchans	List of electrodes/channels to show in the main GEMS figure and to be considered for processing
	initializeTime	Starting time of viewing signals in the main GEMS figure and hence the starting time for the processing data
	dataSegmentLength	Length of the viewing window in main GEMS figure and hence the duration of the processed data
	minSliderMove	Minimum amount slider can move by in main GEMS figure
	maxSliderMove	Maximum amount slider can move by in main GEMS figure
	yscaleVal	Sets loose or tight y-axis (controls vertical space occupied by signal in each subplot in the main GEMS figure)
	stackplotVal	Stack plots or plot them as grid in main GEMS figure
	cols	Number of columns of subplots in main GEMS figure
	FiltType	Filter type to apply on raw signal
	FiltLims	Filter limits to apply on raw signal
	FiltPoly	Polynomial order for filtering
	FiltWind	Filtering half window size
	Nxtnd	Number of samples for extended filter
	basesubMethd	Baseline removal method
	lvld	Level of wavelet decomposition
	baseWindSize	Half window size for baseline removal
	zerolvl	Number of scales to reconstruct (largest scales) for baseline subtraction
	sortchans	Sort channels
	ShowFFT	Controls whether to show figure with FFT of signals displayed in main window
	FFTlims	Controls the frequencies for which the FFT

		is computed
	Tau	SOBI integer time lags
	sobipkLbl	Controls whether to label peaks in SOBI components frequency spectra
	pmaxVal	Threshold for peak labelling
	useSymm	Controls whether to use the symmetric version of SOBI
Detection Parameters	*thresh*	Multiple of rms noise estimate
	etamax	Spikes too large in signal will not be declared, multiple of rms noise
	refractory	Refractory period between slow waves
	smoothWinSz	Smoothing window width of sneo signal
	method	Detection method (simple|sneo|differ)
	DownDeflOnly	Detect downward deflections only using peak detector kernel x detection sig
	UpDeflOnly	Detect upward deflections only
	Nedge	Size of peak detector kernel
	rmhw	Half width of running median threshold detection
	baselineMetric	Metric used to define the baseline noise level in a signal (mabdev|rms|std)
	defSpikeTime	Determines how to define the activation time w/in FEVT window (min|maxdiff)
	checkEdge	Check putatitive events near boundary of finite data segment using local min criterion
	Tbc	Disallow spikes within first 15 samples = 1/2 second. Prevents an artificial "spike" in SNEO from being recognized
	mxstat	Intermediate output of FEVT detection signal
	minEventDur	Minimum event duration to be considered a spike
	ampThresh	Empty means no amplitude threshold criteria applied
	derivThresh	Empty means no deriv threshold criteria applied
	polyorder	Order of polynomial for baseline fitting to local spike waveform
	verbose	Option to print more infomartion during detection main matlab cmd prompt.
	useMex	Option sets whether to use mex implementation in FEVT detection callback.
	amplthresh	Threshold to determine if a peak or trough has been established
	window_half_length	Half window length for amplitude detection
Partition Parameters	*DTMAX*	Sets delta T allow queued point membership into growing region
	doBaylyEst	Do Bayly curve to estimate activation time

		of next locations event
	FitOrder	2 = 2nd order bayly surface; 3 = 3rd order surface
	N_FIT_POINTS	Use this many points in the running estimate of the next points activation time
	N_BAYLY	Turn on bayly estimation only after this many points are in the cluster (must be >=6)
	SEED_ELEC	[] lets getSeed() programmitcally choose based off of CM of Nevents criterion
	manualCluster	Manual or automatic clustering.
Map Plotting Parameters	*plotTimeInterp*	Determines type of interpolation to plot isocrhones (inverse|nearest|v4|bayly|off) `WARNING: *nearest* and *v4* produce misleading interpolations`
	isoLabels	Controls printing of text values on contour lines
	minPlotRatio	The minimum ratio of detected events per wave number required to plot maps
	plotHlafElecSpace	Plots ATs at half electrode spacing or on electrodes
	contourOnly	True: plot contour plot only for the AT maps (no surfaces). False: plot contour surface plots for the AT maps. **(Disconnected at the moment)**
	condBlockThresh	Velocity threshold value (%) used to determine if a conduction block exits. E.g., if the parameter is set to 0.3, this indicates that a conduction block will exist if the velocity between adjacent electrodes is less than 30% of the average velocity value for that wave number.
	setMapLimits	True: user inputs limits manually for maps when exporting to image file.
	plotElecGrid	Determines whether to plot white/black dots to mark electrode grid
Flash Movie Parameters	*flashlightTlag*	Flashlight movie time-lag window
	flashlightST	Flashlight movie start-time
	flashlightET	Flashlight movie end-time
	flashlightDT	Flashlight movie time step-size
	flashlightRefracOnly	Show only the trailing refractory tail. "Leading" wavefront edge will not be shown
	flashlightFPS	Frame per second playback speed
	flashlightBW	Use black squares on white background or vice versa
	flashlightUseCycles	Use REGROUPS partitioned cycles to make movies or all detected events

	flashlightUseColors	Use colours for the movie
	flashlightManualColors	Set manual colours for the movie
	flashlightMarkChanls	1: Does not mark channels, 2: Marks channels with no ATs in the selected time period, 3: Marks channels with no detected ATs
	flashlightWithInfo	True: splits the screen of the movie to 2 sections, half of it will contain user specified image and information and the other half is the movie. If user selects the BW movie, the default will be red on either white or black. False: default setting of movie generation, no extra information.
	flashlightDispRealTime	True: Display real time on top of the movie. False: Offset the display start time by OffsetTime parameter.
	flashlightOffsetTime	Offset the display start time
	flashlightSelectPatch	True: User to select a patch from the electrode matrix to create the movie. False: Entire electrode matrix is considered for creating the movie.
	flashlighTopLeftElec	Top left electrode number of the selected patch, only used if *flashlightSlectPatch* parameter is set to true.
	flashlightPatchRows	The number of rows of the selected patch, only used if *flashlightSlectPatch* parameter is set to true.
	flashlightPatchCols	The number of columns of the selected patch, only used if *flashlightSlectPatch* parameter is set to true.
	flashlightMovieFileType	'avi' saves to avi file, 'tif' saves frames to tif files which can then be combined to make a movie.
	flashlightInterpTimes	Interpolate unmarked sites in each frame, similar to interpolating isochrone plots

20 For developers (open source only)

If you are changing some code in a certain function and want to know where the function is getting called from, follow the following instructions:

1. Ensure you have perl installed in your machine. For instructions on how where you can obtain that please refer to Section 2.
2. Add the path to your GEMS folder **addpath(genpath('path/directory'))**.
3. Run funcTree.m. This might take some time, as it is searching all the relevant functions used in GEMS and how they are connected. Once finished a figure as shown in Figure 22 will pop-up.
4. All functions found are listed alphabetically (list is sorted by taking functions which start with capital letters first) in the *Functions list* listbox. These functions are the functions inside GEMS folders, excluding the analysis_scripts and obsoleteFunctions folders.
   NOTES:
   - The obsoleteFunctions folder contains functions which are no longer used but we keep them just in case. If you want to add any functions to this folder, please ensure that the entire .m file is commented so that it will not be considered in the path.
   - The analysis_scripts folder contains functions which we are not really interested in, we will eventually clean it up.
5. As you click on the function you are interested in in the *Functions list* listbox, the path to that function gets displayed in the *Path* editbox at the bottom of the figure.
   NOTE: you can scroll through the function names by hitting the letter you are interested in while you are in the *Functions list* listbox. For example, if I want to find zchan function, I can just hit 'z' on my keyboard and it will take me to the function that start with z. As you keep hitting 'z' the selection moves to the next function that starts with z.
6. Once you select a function from the *Functions list* listbox, the sub-functions will be listed in the *Sub-functions* listbox. The sub-functions are the functions found inside the chosen function in the *Functions list* listbox.
7. Once you select a function from the *Functions list* listbox, a list of the functions where the selected function is called from will be displayed in the *Called-from functions* listbox.
8. If you click on any of the functions in the *Sub-functions* or *Called-from functions* listbox, the function file will open up in MATLAB. This is to assist you change the functions.
9. The total number of functions used in GEMS (again excluding the analysis_scripts and obsoleteFunctions folders) is displayed in the *Total number of functions* text box.
10. The total number of code lines used in GEMS (again excluding the analysis_scripts and obsoleteFunctions folders) is displayed in the *Total number of lines* text box.

NOTE:
The *Sub-functions* or *Called-from functions* listboxes might contain more than the realistic used functions. This is a limitation of the search, because sometimes we have parameters the same name as a function, so the search doesn't know any better!!!.

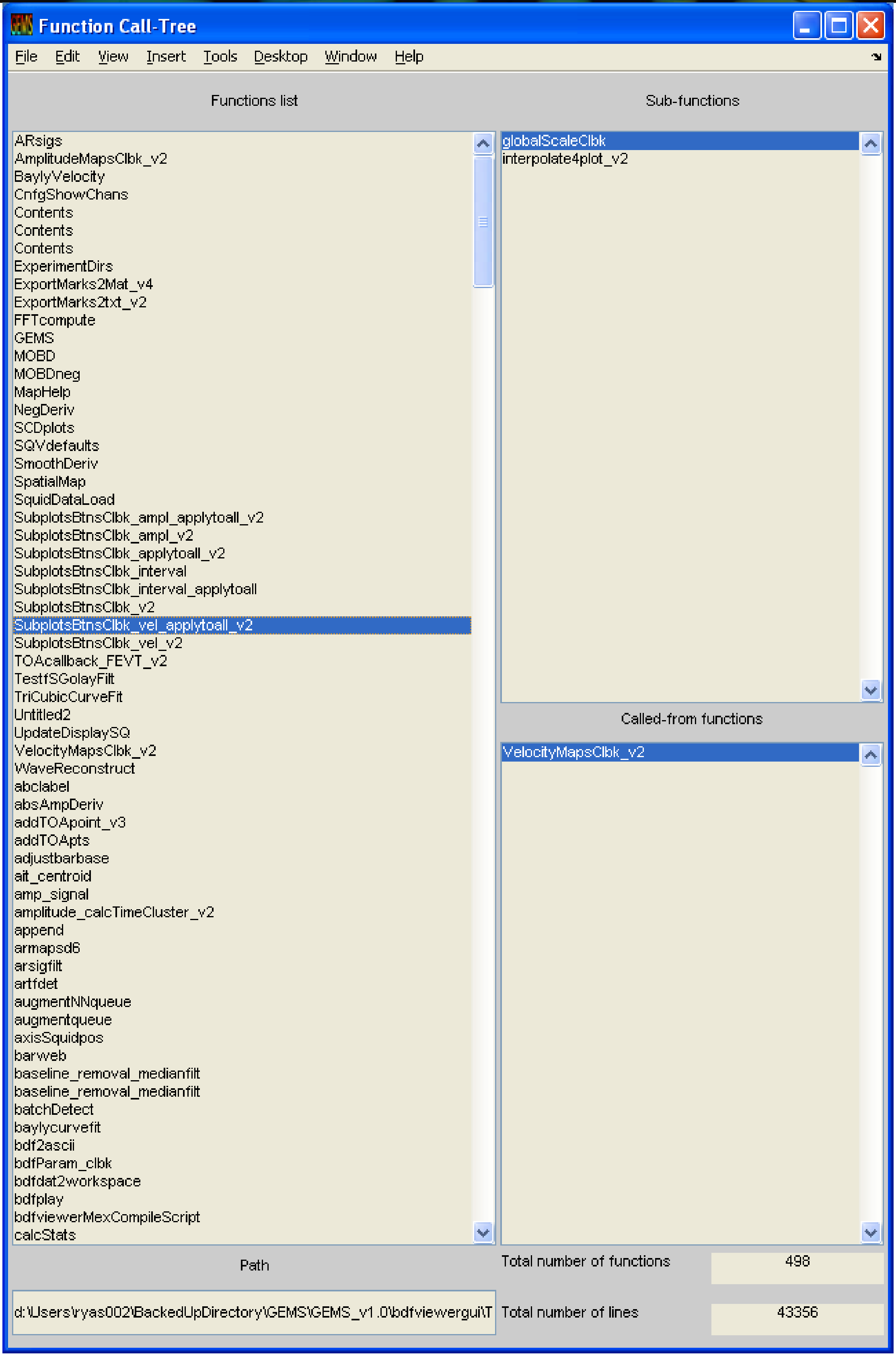

Figure 22: Function call-tree.

21 Back-end information

21.1 Clustering algorithm

This algorithm is used to group detected ATs from different channels or cluster them under the same event number. The algorithm basically implements a region growing/clustering algorithm. The criterion for membership in a cluster is that the activation time under investigation must not defer more than DTMAX (user parameter) than the estimated time. The estimated time is computed as the mean of the ATs for the first few points. After the regions becomes large enough (also a user parameter, N_BAYLY), the estimated time is computed by using the points already in the cluster to generate the coefficients for a Bayly surface:
f(x,y) = a*x^2 + b*y^2 + c*x*y + d*x + e*y + f;
then extends the surface to estimate the time at the point under investigation.
(c.f. *Bayly et al. Estimation of Conduction Velocity Vector Fields from Epicardial Mapping Data. IEEE Trans. Biomed Eng. Vol 45 No 5 May 1998.)*
The algorithm is known to be sensitive to the "seed electrode", the point at which the cluster is seeded, from which it expands outward.

REGROUPS critical parameters

DTmax – Difference between actual and estimated AT. This should be set to about 10-25% of the (expected/measured) slow wave propagation cycle period. For example, if a gastric slow wave period is 20 s (3pm), then DTmax should be between 2-5 s. Tradeoff: setting a lower value increases likelihood of a false positive being included in a cluster; setting value too high increases chances of misclustering an activation time that is part of another slow wave. In GEMS this parameter can be found under the **Parameters Figure – Partition parameters – DTMAX**.

Ncrit—Determines minimum number of points in a cluster at which polynomial curve fitting for estimation is "turned on." Ncrit must be > 6. In practice, we have found a slightly higher value (8-16) works well, and that the algorithm is fairly insensitive to this parameter. Tradeoff: Lower value of Ncrit increases likelihood of local over-fitting, leading to poor estimation of ATs; higher value of Ncrit turns on polynomial fitting at a later time, making for poorer estimation of ATs on a global scale. In GEMS this parameter can be found under the **Parameters Figure – Partition parameters – N_FIT_POINTS**.

21.2 FEVT detection

ATs are detected based on a Non-linear Energy Operator (NEO) method. The algorithm implemented here computes either the energy as a function of time for an electrode signal V(t), or of the first difference of the signal, diff(V(t)). Then the energy is analyzed to find times at which it is statistically "very high." The basis of the idea is that serosal records of gastric slow wave events that have a relatively steep decrease at the onset, followed by a slow recovery. The NEO operator is very sensitive to relatively steep drops in a signal, as it is proportional to $A^2\omega^2$ (the amplitude squared multiplied by the frequency squared).

Brief description of AT detection parameters:
Method (sneo | differ): defines the method to use to detect ATs.

sneo: computes the energy as a function of time, E{V(t)}, of a signal (electrode).

diffE: computes the energy of the first difference of the signal as a function of time E{diff(V(t))}

Detection Threshold: specifies that ATs are (potentially) detected at times for which the energy exceeds detectionThreshold*estimated RMS noise in signal.

Refractory time (sec): specifies how far apart large "bumps" in the energy must be separated to be considered separate slow wave events. I have found that values in the range of 3-5 seconds work well.

Boundary Check (sec): Because finite data segments can have artificially large values for the energy at the edge, check to see if we believe these are real slow wave events. Any putative slow wave occurring within "BoundaryCheck" seconds of either side of the segment are subjected to a further test for accuracy.

Smoothing window(sec): smooths the energy spectrum with a moving boxcar window of "Smoothing window" seconds.

FEVT critical parameters (optimized for gastric slow waves)

η (threshold multiplier): multiplies the "noise estimate" (running median) to set the actual event threshold level. A value between 4 – 6 is typical. Tradeoff: lower eta increases sensitivity, but decreases positive-prediction value; and vice versa. Performance is dependent upon actual value set. In GEMS this parameter can be found under the **Parameters Figure – Detection parameters – thresh**.

τ_w (running median window half-width): determines time-window over which running median is calculated. In practice, this should be several times larger than the "active waveform" timescale (i.e., deflection and recovery to baseline). Performance is essentially independent of this parameter otherwise. For gastric signals, 15-30 sec is recommended; Larger values lead to slower computation. In GEMS this parameter can be found under the **Parameters Figure – Detection parameters – rmhw**.

Tr (refractory period): specifies the minimum time between two putative ATs. Should be set long enough to account for fractionated waveforms, but short enough to avoid grouping two independent ATs into a single event. Recommended value: 6 s (for gastric). In GEMS this parameter can be found under the **Parameters Figure – Detection parameters – refractory**.

N_{edge} (edge detector kernel width): Integer number of samples in edge detection kernel. Helps identify deflection direction. Should be set to match the time scale of active waveform's main deflection. Therefore, when choosing Nedge, be mindful of the sampling frequency! A value of 1 s (= 30 samples for 30 Hz sampling) works well for gastric slow wave. In GEMS this parameter can be found under the **Parameters Figure – Detection parameters – Nedge**.

22 References

JC Erickson, G O'Grady, P Du, C Obioha, W Qiao, WO Richards, LA Bradshaw, AJ Pullan, and LK Cheng. 'Falling-edge, variable threshold (FEVT) method for the

automated detection of gastric slow wave events in high-resolution serosal electrode recordings'. *Ann. Biomed. Eng.* **38**(4):1511-1529, 2009.

JC Erickson, C Obioha, A Goodale, LA Bradshaw, and WO Richards. 'Detection of Small Bowel Frequencies from Noninvasive Biomagnetic Measurements'. *IEEE Trans. Biomed. Eng.* **56**(9):2181-2189, 2009.

S Mukhopadhyay, and GC Ray. 'A new interpretation of nonlinear energy operator and its efficacy in spike detection'. *IEEE Trans. Biomed. Eng.* **45**(2):180-187, 1998. (http://ieeexplore.ieee.org/ielx5/10/14396/00661266.pdf?arnumber=661266)

JC Erickson, G O'Grady, P Du, JU Egbuji, AJ Pullan, and LK Cheng. 'Automated gastric slow wave cycle partitioning and visualization for high-resolution activation time maps'. *Ann. Biomed. Eng.* **39**(1):469-483, 2010. Doi: 10.1007/s10439-010-0170-8.

23 Contributors

Dr. Jonathan C Erickson (GEMS founder/developer)
Dept of Physics-Engineering
Washington & Lee University, Lexington, VA, USA.

Dr. Rita Yassi (GEMS manager/developer)
Department of Engineering Science
The University of Auckland, New Zealand.

Mr. Nira Paskaranandavadivel (GEMS developer)
Auckland Bioengineering Institute
The University of Auckland, New Zealand.

Dr. Gregory O'Grady
Dept of Surgery & Auckland Bioengineering Institute
The University of Auckland, New Zealand.

Mr. Peng Du
Auckland Bioengineering Institute
The University of Auckland, New Zealand.

Dr. Leo Cheng
Auckland Bioengineering Institute
The University of Auckland, New Zealand.

Prof. Andrew Pullan
Dept of Engineering Science & Auckland Bioengineering Institute
The University of Auckland, New Zealand.

Mr. Tim Angeli
Auckland Bioengineering Institute
The University of Auckland, New Zealand.

Dr. John Egbuji
Auckland Bioengineering Institute
The University of Auckland, New Zealand.

Dr. Wim Lammers
Dept of Physiology,
United Arab Emirates University, Al-Ain, UAE.

Ms. Emily Hargrave-Thomas (GEMS developer)
Auckland Bioengineering Institute
The University of Auckland, New Zealand.

Mr. Simon Bull
Auckland Bioengineering Institute
The University of Auckland, New Zealand.

Functions available on File Exchange

GEMS management would like to acknowledge the following authors for their contribution:

Author	Function	URL
**Jianwen Luo**<br>**luojw@ieee.org**	sgsdf_gram_poly.m	http://www.mathworks.it/matlabcentral/fileexchange/5556-savitzky-golay-smoothing-and-differentiation-filter/content/sgsdf_gram_poly.m
**Rasmus Anthin**<br>**rasmus.anthin@surgical-science.com**	rgbconv.m	http://www.mathworks.com/matlabcentral/fileexchange/4265-rgbconv-m
**Jaco de Groot**<br>**j.groot@ucl.ac.uk**	outlier.m	http://www.mathworks.com/matlabcentral/fileexchange/11106
**Aslak Grinsted**<br>**ag@glaciology.net**	samexaxis.m	http://www.mathworks.com/matlabcentral/fileexchange/7169-samexaxis-nice-subplots-with-same-x-axis
**Aslak Grinsted**<br>**ag@glaciology.net**	parseArgs.m<br>subaxis.m	http://www.mathworks.com/matlabcentral/fileexchange/3696-subaxis-subplot/content/parseArgs.m<br>http://www.mathworks.com/matlabcentral/fileexchange/3696-subaxis-subplot/content/parseArgs.m
**Don Orofino**<br>**don@mathworks.com**	MoviePlayer (Folder)	http://webscripts.softpedia.com/script/Scientific-Engineering-Ruby/Signal-Processing/Interactive-MATLAB-Movie-Player-34707.html
**Christopher Torrence**<br>**chris@rsinc.com**<br>**Gilbert P. Compo**<br>**compo@colorado.edu**	wavelet_matlab (Folder)	http://atoc.colorado.edu/research/wavelets/
**Peder Axensten**<br>**peder@axensten.se**	waitbar.m	http://jupiter.ethz.ch/~kausb/perplith_files/waitbar.m
**Toby Driscoll**<br>**driscoll@udel.edu**	unplot.m	http://www.mathworks.com/matlabcentral/fileexchange/2831
**Chris Cannell**<br>**ccannell@vt.edu**	uigetfile2.m,<br>uigetdir2.m	http://www.mathworks.com/matlabcentral/fileexchange/9254-uigetfile2<br>http://www.mathworks.com/matlabcentral/fileexchange/9521-uigetdir2
**Ben Barrowes**<br>**barrowes@alum.mit.edu**	suplabel.m	http://www.mathworks.com/matlabcentral/fileexchange/7772-suplabel
**Evan Brooks**<br>**evan.brooks@wpafb.af.mil**	scrollfigdemo.m	http://www.mathworks.com/matlabcentral/fileexchange/5253-scrolling-figure-demo/content/scrollfigdemo.m
**Ohad Gal**<br>**ohad_gal@amat.com**<br>**Steve Hoelzer**<br>**shoelzer@gmail.com**	progressbar.m	http://www.mathworks.com/matlabcentral/fileexchange/3607-progressbar
**Sergei Koptenko**	local_ext_mod (Folder)	http://www.mathworks.co.uk/matlabcentral/fileexchange/3170-local-min-max-nearest-neighbour
**Jan Simon**	fSGolayFilt.m	http://www.mathworks.com/matlabcentral/fileexchange/5661
**John Iversen**<br>**iversen@nsi.edu**	freezeColors.m,<br>unfreezeColors.m	http://www.mathworks.com/matlabcentral/fileexchange/7943-freezecolors-unfreezecolors

**Ian Howat**<br>**ihowat@gmail.com**	filnans.m	http://www.mathworks.com/matlabcentral/fileexchange/15590-fillnans
**Frederic Moisy**<br>**moisy@fast.u-psud.fr**	fileseries (Folder)	http://www.mathworks.com/matlabcentral/fileexchange/12180-fileseries-rename-renumber-files-recursive-directories/all_files
**Blair Greenan**<br>**greenanb@mar.dfo-mpo.gc.ca**	colorbarf.m	http://www.mathworks.com/matlabcentral/fileexchange/1135
**Carlos Adrian Vargas Aguilera**<br>**nubeobscura@hotmail.com**	cm_and_cb_utilities (Folder)	http://www.mathworks.com/matlabcentral/fileexchange/24371-colormap-and-colorbar-utilities-sep-2009
